# Light Emission and Conductance Fluctuations in Electrically Driven and Plasmonically Enhanced Molecular Junctions

**DOI:** 10.1021/acsphotonics.4c00291

**Published:** 2024-06-06

**Authors:** Sakthi Priya Amirtharaj, Zhiyuan Xie, Josephine Si Yu See, Gabriele Rolleri, Konstantin Malchow, Wen Chen, Alexandre Bouhelier, Emanuel Lörtscher, Christophe Galland

**Affiliations:** † Institute of Physics, Ecole Polytechnique Fédérale de Lausanne (EPFL), CH-1015 Lausanne, Switzerland; ‡ Laboratoire Interdisciplinaire Carnot de Bourgogne CNRS UMR 6303, Université de Bourgogne, 21000 Dijon, France; § IBM Research Europe—Zurich, Säumerstrasse 4, CH-8803 Rüschlikon, Switzerland

**Keywords:** molecular junctions, fluctuating atom-molecular contacts, inelastic electron tunneling, combined transport and optical spectroscopy

## Abstract

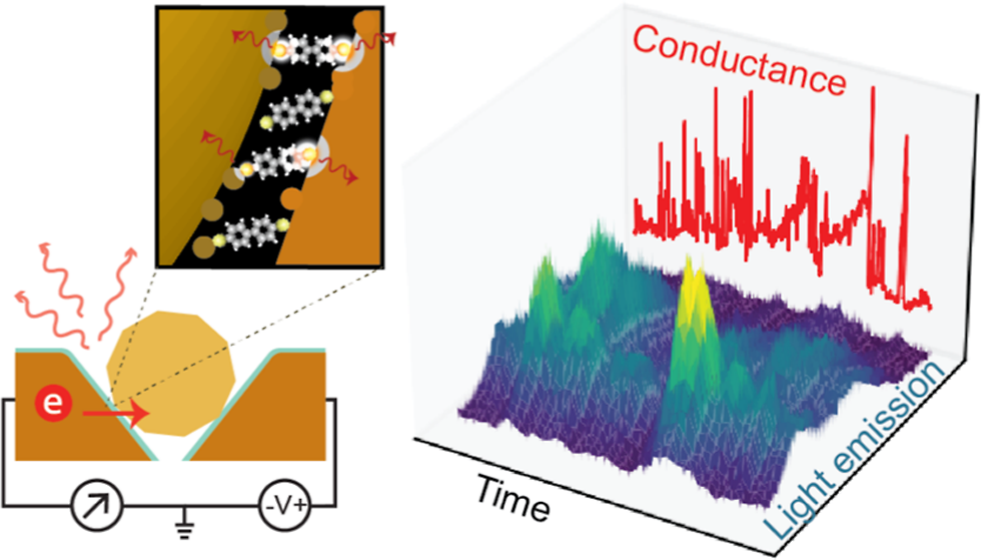

Electrically connected and plasmonically enhanced molecular junctions combine the optical functionalities of high field confinement and enhancement (cavity function), and of high radiative efficiency (antenna function) with the electrical functionalities of molecular transport. Such combined optical and electrical probes have proven useful for the fundamental understanding of metal–molecule contacts and contribute to the development of nanoscale optoelectronic devices including ultrafast electronics and nanosensors. Here, we employ a self-assembled metal–molecule–metal junction with a nanoparticle bridge to investigate correlated fluctuations in conductance and tunneling-induced light emission at room temperature. Despite the presence of hundreds of molecules in the junction, the electrical conductance and light emission are both highly sensitive to atomic-scale fluctuations—a phenomenology reminiscent of picocavities observed in Raman scattering and of luminescence blinking from photoexcited plasmonic junctions. Discrete steps in conductance associated with fluctuating emission intensities through the multiple plasmonic modes of the junction are consistent with a finite number of randomly localized, point-like sources dominating the optoelectronic response. Contrasting with these microscopic fluctuations, the overall plasmonic and electronic functionalities of our devices feature long-term survival at room temperature and under an electrical bias of a few volts, allowing for measurements over several months.

## Introduction

Plasmonic nanocavities with extreme light confinement allow electromagnetic interactions with few or even single molecules to be studied and tailored.^1,2^ They can be electrically connected,^3^ resulting in molecular junctions that provide opportunities to measure both molecular transport^4−7^ and plasmonically enhanced optical signals. Such combined plasmonic and electrical experiments on molecular junctions are useful for a fundamental understanding of molecular properties and metal–molecule interfaces and for various applications including high-speed nanosized electronics.^8−10^ For instance, electrical transport and spectroscopy of metal–molecule–metal junctions reveal quantum properties originating from structural and chemical rearrangements, observable even at ambient conditions.^5^

While surface-enhanced Raman scattering (SERS) is the most popular spectroscopic technique employed on molecular junctions,^11−15^ electrically driven light emission is a more direct probe of the metal–molecule interface involved in both molecular transport and plasmon-enhanced light emission.^16−21^ This broadband emission resulting from inelastic electron scattering can be understood as originating from the optical-frequency quantum shot noise of electrons tunneling across the potential barrier formed by the molecular junction.^22−25^ Such inelastic electron tunneling (IET) emission can efficiently couple to the plasmonic modes of the host nanocavity.^26^ The electrical and optical signals of these nanoscale devices contain information about the metal–molecule contacts as demonstrated by tip-enhanced Raman spectroscopy and scanning-tunneling microscopy (STM) experiments, including STM-induced luminescence.^20,24,27−31^ Probing an individual molecule usually requires complex systems operating at ultrahigh vacuum and/or cryogenic temperatures.

Here, we investigate single-molecule fluctuations within nanoscale plasmonic molecular junctions (PMJs) formed around a self-assembled monolayer (SAM),^6,32−34^ which operate at room temperature. A SAM of thiol-terminated molecules is created on top of two lithographically defined electrodes bridged by a few nanoparticles. The idea of bridging nanoparticles between electrodes coated with SAM was proposed by Amlani et al. in 2002.^35^ But for a long time, only the electrical properties of the molecular junctions were investigated. Kern et al. used a similar approach to trap nanoparticles with native ligands between predesigned antenna structures to create a sub-nm gap and demonstrate electrically driven plasmonic emission by IET.^26^ The ligand molecules there merely played the role of an insulating spacer. Inspired by these approaches, we created electrodes with slanted sidewalls that are optimized for the collection of the optical signal once a nanoparticle falls in between to form hybridized plasmonic resonances.^36^ A similar design was simulated, tested, and implemented as a dual-band nanoantenna in a previous study.^37^ These nanocavity junctions provide significant plasmonic enhancement that allows for fast optical measurements to probe atomic-scale fluctuations at the metal–molecule contact through its impact on light emission mediated by the tunneling of electrons across the gap. The junctions can be studied over weeks and months, allowing us to thoroughly investigate the impact of atomic fluctuations in the junction on both its photoemission and electrical transport characteristics. The devices continue to be dominated by molecular transport until they get damaged under too high applied voltage (several volts), too large optical powers (mW/μm^2^), or by electrostatic discharge (ESD).

Our main finding is that a few randomly switching current conduction channels appear to control the macroscopic behavior of the device, despite the large number of molecules acting as a spacer. This microscopic dynamics is evidenced by discrete jumps in the light emission spectrum, as well as joint fluctuations of emission intensity and conductance that are consistent with a minimal model of fluctuating atom-molecule contacts. Similar point-like emission was recently evidenced in a large-area SAM tunnel junction between gold and eutectic gallium–indium alloy (EGaIn) contacts^38^ where it was attributed to conformational changes in the molecule due to the excitation of vibrational modes, and no such blinking was observed in the current through the junction.^39^ In ref (38) the resonance and polarization of the point-like plasmon source were modified by the applied bias voltage. With our junctions, we find no such shift in the plasmonic resonance with applied voltage. The plasmonic response is fixed by the nanoparticle–electrode cavity and is excited by electron tunneling through the junction, and we observe correlated blinking in both the light emission and conductance. Our results therefore suggest that a phenomenology similar to that underlying the occurrence of picocavities in SERS,^40,41^ of blinking in gold nanojunction photoluminescence,^42^ and of flickering in electronic Raman scattering^43^ can also be driven by electrical bias. At present, investigation of metal atom movements in molecular junctions relies mostly on STM or break-junctions in the regime of quantum point contact.^20,44−46^ We demonstrate that a bulk nanoparticle–electrode system can capture fluctuations in both the conductance and light emission and extend the optoelectronic investigation of quantum-point contacts in STM/break-junctions to a completely different regime of conductance, junction geometry, and operation conditions.

## Results and Discussion

**Figure 1:**
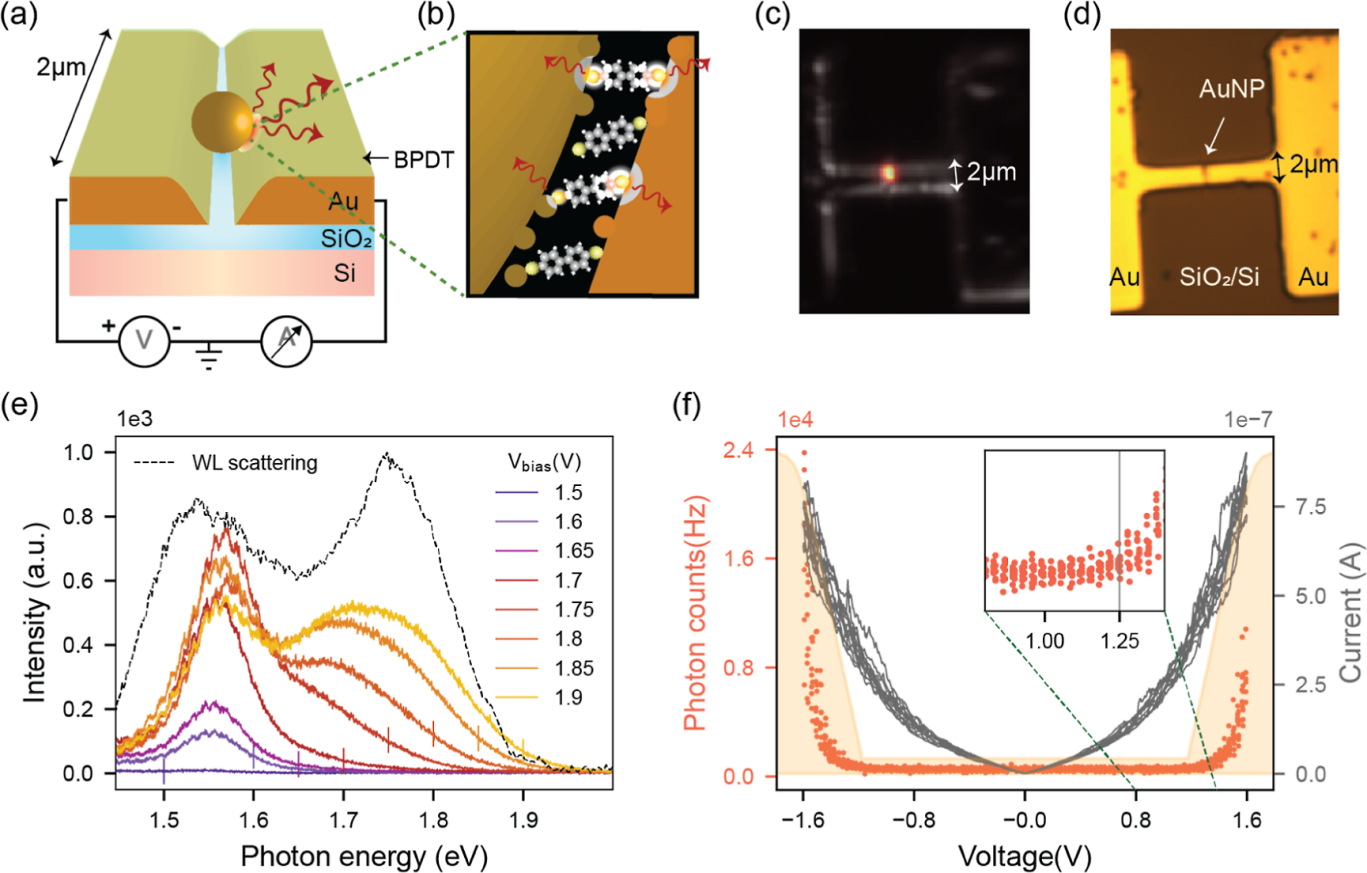
(a) Schematic illustration of the PMJ. (b) Illustration of atomic fluctuations and light emission at the metal–molecule–metal contacts. (c) Grayscale dark field image overlaid with the colored light emission image under 1.6 V bias for a typical PMJ. (d) Corresponding bright field image. (e) Light emission spectrum for various dc voltages with 20 s exposure time. The vertical lines mark the onset of overbias emission. Dotted lines represent the dark-field scattering spectrum from a white light source. (f) Current vs voltage characteristics of the junction (gray curve) and simultaneously collected photon counts (red curve) as a function of applied voltage. The shaded background represents the quantum efficiency of the detector with voltage values translated to photon energy in eV. Inset: zoomed-in view with the vertical line showing the onset of photon detection.

The PMJ is formed by 150 nm gold nanoparticles (from a citrate-stabilized colloidal suspension) bridging two gold electrodes that are previously functionalized with a SAM of biphenyl-4,4′-dithiol (BPDT) molecules (Figure 1a). One or few gold nanoparticles are trapped in the gap and establish electrical contact (see Supporting Information Section S1 for sample fabrication and experimental methods). Each nanoparticle linker forms in fact two junctions in series, but one of them typically dominates the series resistance and experiences most of the voltage drop, as will be discussed below. The device conductance *G* after fabrication falls in one of the following ranges: (1) short-circuited contact where *G* ∼ 10^2^·*G*_0_ where *G*_0_ = 2*e*^2^/*h* is the quantum of conductance; this occurs in particular in junctions fused by ESD. (2) Open-circuited contact where *G* < 10^–6^·*G*_0_; this occurs due to several reasons: (i) there is no nanoparticle bridging the gap; (ii) the size of the nanoparticle is smaller than the size of the gap; and (iii) the nanoparticle does not establish successful contact with the electrodes on both sides. (3) Molecular contact where 10^–5^·*G*_0_ < *G* < 10^–1^·*G*_0_. Sometimes an initially open-circuited junction can be brought into molecular contact by applying several volts. We also note that junctions featuring the largest conductance (*G* > 2 × 10^–3^·*G*_0_) cannot be studied for light emission, as under voltages above 1.2 V, the corresponding current exceeds the damage threshold of the device, which is typically a few μA and above which irreversible changes occur (see Supporting Information Section S3.3).

The nanogaps formed by the SAM between the metallic parts have a width of around 1 nm that depends on the length of the molecules and their orientation with respect to the gold surfaces.^47^ They support localized plasmonic resonances that offer extreme light confinement and good radiative efficiency^36^ and allow to efficiently read out the plasmonically enhanced optical signals. When a voltage bias is applied across this device, the PMJ emits light originating from the optical-frequency shot noise of electrons inelastically tunneling across the junction through the potential barrier as discussed below.^22−26^ An image of light emission from one PMJ overlaid with a dark-field scattering image is shown in Figure 1c, with the corresponding bright-field image shown in Figure 1d. The emission couples to the plasmonic modes of the cavities formed by the nanogaps between the nanoparticles and electrodes and can be observed in the far field.

The light emission spectrum of BPDT junctions for different dc bias voltages and an exposure time of 20 s is shown in Figure 1e. Note that there is a cutoff observed in the light emission spectrum at an energy close to the value of eV (V is the applied voltage and e is the electron charge), as indicated by the vertical line in Figure 1e. This is also asserted in Figure 1f, where the photon count rate increases nonlinearly beyond a threshold of about 1.25 V that matches the spectral response of the silicon detector having a cut-on around this photon energy. While we expected the electrode–molecule–nanoparticle geometry to form two identical molecular junctions in series, the fact that the energy cutoff matches well with the applied voltage suggests that most of the voltage drops across one of the two junctions (consistent with previous observations of similar junctions^26^) and the light emission happens through a single-electron inelastic tunneling process.

Some overbiased light emission is also observed in our PMJs, with photon energies higher than the applied voltage (times the electron charge). This was previously demonstrated in STM junctions,^27,48−51^ electromigrated junctions,^17,19,52^ mechanical break junctions^53^ and more recently memristive junctions.^54^ This emission was either attributed to hot carriers or coherent multielectron scattering processes. In our experiment, the excess photon energy is in agreement with a moderate increase in the electron temperature in the junction^545^ (see Section S5 in Supporting Information).

The two peaks in the spectra are attributed to plasmonic modes of the junction, also seen in the dark-field scattering spectrum (dotted lines) collected using a tungsten lamp. The nanocavity design is adapted from a previous study involving a nanoparticle-in-groove dual resonant cavity^37^ that exhibits several resonances at visible and near-infrared wavelengths. There is no evidence of hot electron emission in our system (see Section S5), and the emission is found to be shaped by the plasmonic response as discussed in Section S4 of Supporting Information. We refer to ref (37) for a simulation of the visible plasmonic modes of a similar structure. The plasmonic modes are progressively populated upon increasing the voltage, as observed with the onset of emission from a plasmonic mode at higher energy for voltages above 1.7 V.

**Figure 2:**
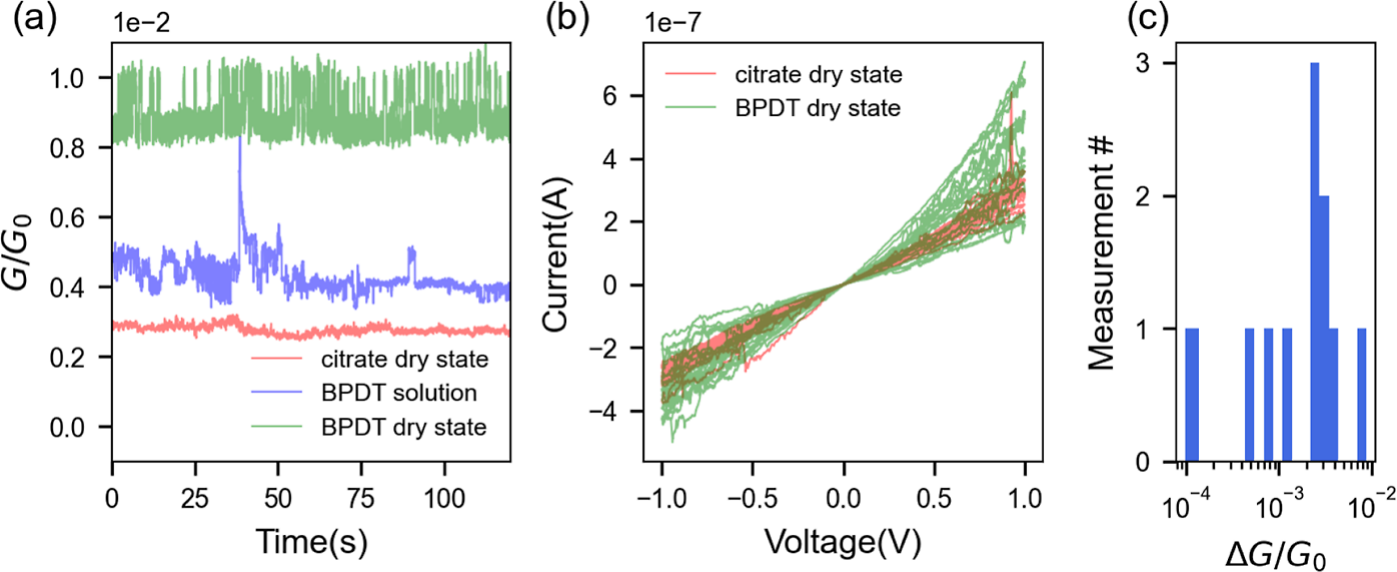
(a) Conductance of a particular PMJ with citrate (red) spacer in a dry state, during deposition of BPDT molecules (blue) and after deposition of the BPDT molecules (green), measured under 5 mV bias. (b) Current–voltage characteristics of the PMJ before and after deposition of BPDT molecules. (c) Magnitude of conductance changes evaluated from several measurements where clear intermittent blinking was observed. The data are collected from 6 distinct devices (see Figure S5 in Supporting Information).

**Figure 3:**
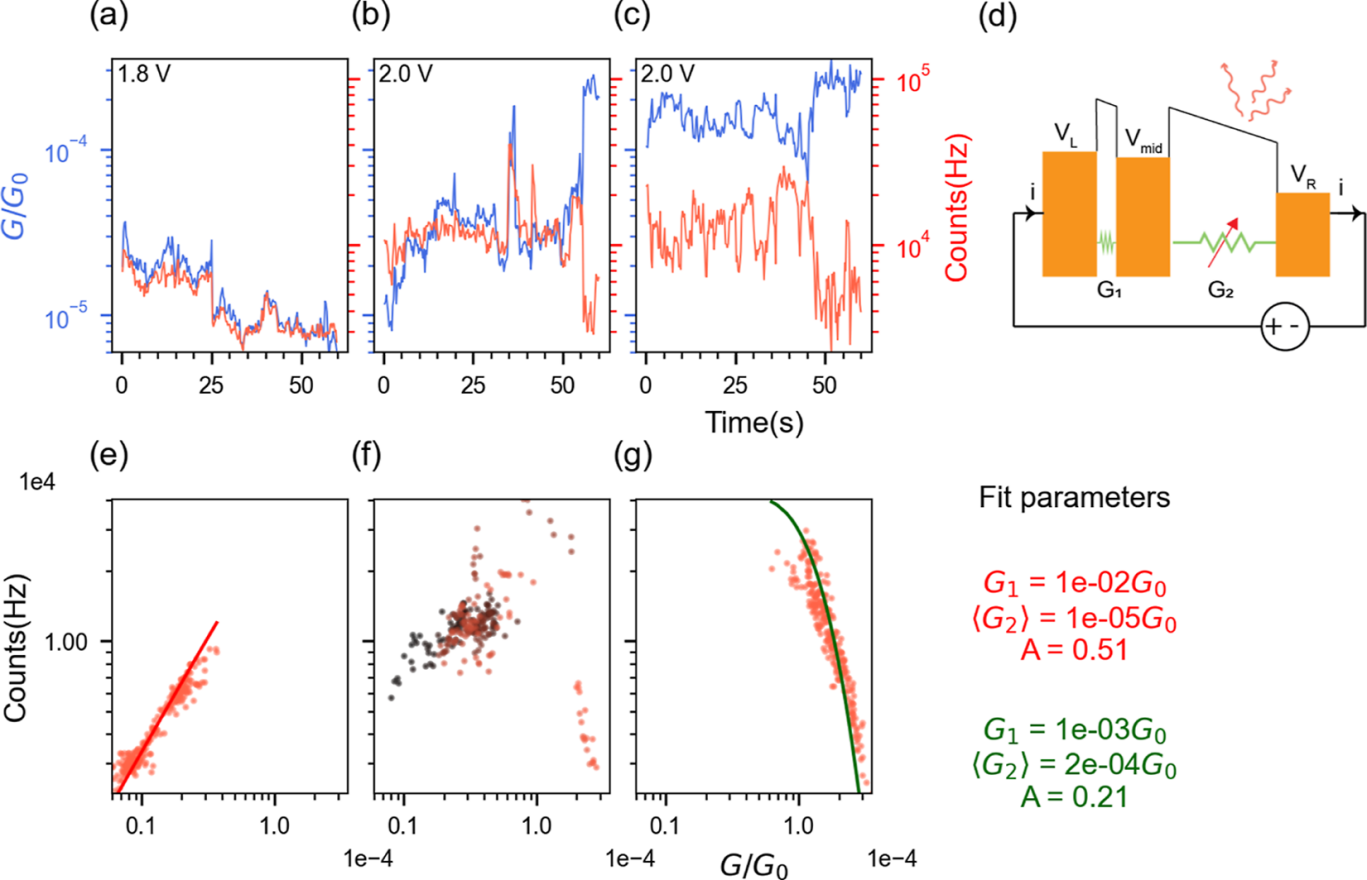
(a–c) Conductance (blue lines) and simultaneously measured photon counts (red lines) for a particular PMJ. Both data are summed into 500 ms time bins. (d) Model of the device with fluctuating conductance. (e–g) Corresponding correlation plots of the data from (a–c) display the switching between positive and negative correlations (see transition within (f)—the data points are color coded from black to red, representing the progression of time). The solid lines in (e) and (g) are from the model discussed in the text, with the corresponding parameters indicated on the right panel.

**Figure 4:**
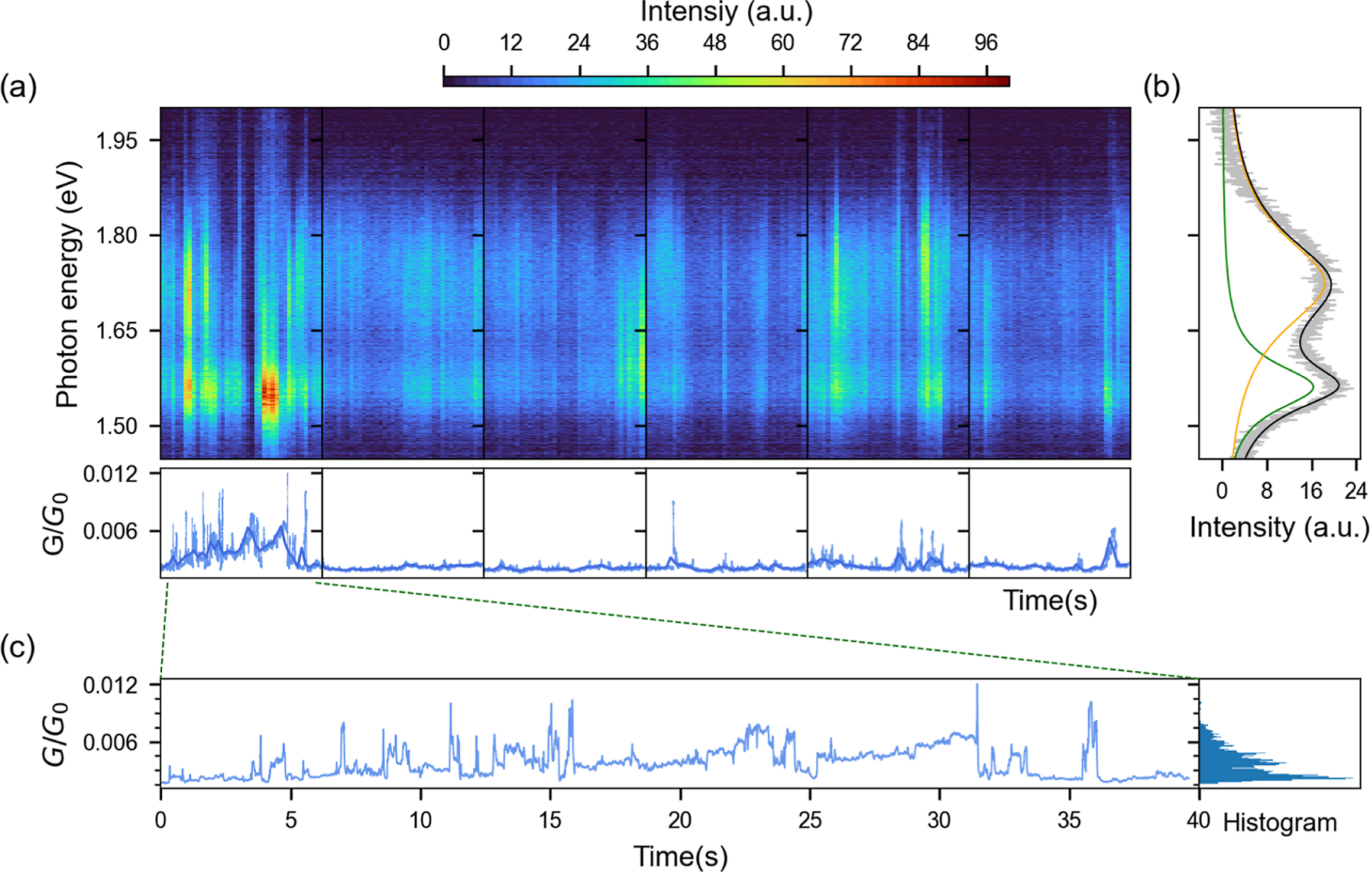
(a) Fluctuations in the light emission spectra (top panel) and the electrical conductance *G*/*G*_0_ (bottom panel) collected simultaneously from the same PMJ. A constant dc bias voltage of 1.9 V was applied, spectra were collected with 1 s exposure time, and the conductance data were measured simultaneously at a 2 kHz sampling rate. (b) The gray curve represents the average of all the spectra in (a), and the black solid line illustrates the fit obtained from two Lorentzian peaks (orange and green lines). (c) A subset of electrical transport data from (a) showing discrete jumps in the conductance. The histogram of the conductance data is shown in the right panel, suggesting a quasi-continuum of conductance states.

Despite using SAMs as molecular-ensemble contacts, we typically find conductance values that are rather in the range of few-molecule junctions.^7,55^ We, therefore, expect that the resulting light emission from IET originates from one or few spatially localized emitters (i.e., point sources) whose localization is varying over time across the electrode gap, as pictured in Figure 1b. We now study the direct optical and electrical signatures associated with the dynamic localization of these point-like emitters as discussed in (Figures 2–4).

The current–voltage characteristics (gray curve in Figure 1f) show the typical nonlinear electrical response of a molecular junction.^5^ Given the nanometer size of the molecular spacer, we propose that the main transport mechanism is tunneling. The thiol anchor group of the BPDT molecule forms strong covalent bonds with the gold atoms and the current flow should be mainly mediated by the HOMO levels.^56^ However, since we perform the experiment at room temperature the resonant molecular orbitals of BPDT cannot be identified from the current–voltage characteristics. Also, note that the HOMO–LUMO gap of BPDT molecules is high (3.85 eV^56^), and hence de-excitation through the molecular orbitals is not involved in the light emission process for the voltages considered in this study. There could be additional tunneling contribution from the citrate molecules present in the gap as BPDT may not be replacing all the citrate molecules around the nanoparticles. However, the citrate molecules are weakly bonded to the gold atoms and form an even smaller nanogap between the electrodes.^57^ Pure citrate junctions are characterized in Supporting Information (Section S3.1) which show a much lower yield of conductive junctions compared to the BPDT junctions. The presence of BPDT molecules on the surface of gold readily attracts nanoparticles in the nanogap to bridge the electrodes and establish electrical contact.

In order to further elucidate the nature of the spacers in the PMJ, a progressive addition of BPDT to a native PMJ with a pure citrate spacer is carried out. A PMJ is first formed by depositing citrate-capped nanoparticles in the electrode gap. A solution of BPDT molecules is dropped on the PMJ, and the conductance is monitored simultaneously. We observe that conductance fluctuations become more pronounced when the solution of BPDT is present. After the droplet evaporates, some BPDT molecules are expected to replace the citrate spacer in the nanogap. We find that the dc conductance (Figure 2a) and the current–voltage characteristics (Figure 2b) of the PMJ with BPDT fluctuate more than the initial PMJ with citrate spacer (see also Section S3.2 of Supporting Information). The increased fluctuation of current density in the BPDT junction is attributed to the formation of gold–thiol “staples”. The gold–thiol bond is capable of lifting adatoms on metal surfaces^40,41,58^ and was found to increase the frequency of these events.

Apart from an increase in the average conductance value post-functionalization (that may further evolve over long measurement times, see Supporting Information Section S6), we observe clear telegraph noise that is consistent with a randomly switching contact at the single-molecule level (Figure 2a). The biphenyl molecule’s conductance depends on its conformation^55^ and coordination geometry of the terminal thiols to the gold atoms.^59^ However, the changes in conductance due to these intramolecular phenomena are averaged out at room temperature where the aromatic systems are oscillating around an average position. The large discrete conductance jumps, which can reach an order of magnitude in some cases, rather point to the binding and unbinding of metal–molecule contacts,^60^ the time-scale of which has been found to be on the order of a second at room temperature.^41^ Postselecting conductance measurements from 6 distinct BPDT PMJs at a bias voltage of 5 mV that show clear intermittent blinking, the magnitude of the conductance jump is estimated to be between 1 × 10^–4^*G*_0_ and 8.7 × 10^–3^*G*_0_ (Figure 2c), which is in agreement with the conductance of a single BPDT molecule found in the literature.^61−64^ The conductance variation due to conformational change is much lower in magnitude.^55,65^ Breaking of a single molecular contact mostly occurs at the Au–Au bond which has a bond strength of 1 eV as compared to the Au–S bond strength of 1.6 eV.^66^ Thus, measuring the conductance of the PMJ provides information about fluctuations at the metal–molecule interface. The point-like IET emitters resemble randomly occurring adatom protrusions at metallic nanogaps that can locally affect the plasmonic response. These atomic-scale events are usually probed by laser spectroscopy, mainly by SERS^40^ and tip-enhanced Raman scattering^30^ and more recently manipulated by STM in the regime of quantum point contact.^20,45,46^ Here, we show a novel probe of atomic-scale fluctuations driven by electrical current in a robust PMJ: our data are consistent with the picture of randomly occurring preferential sites for conductance and light emission.

Our PMJs remain functional over weeks and months (see Section S6 in Supporting Information), allowing us to perform in-depth characterization of their dynamic behavior. In particular, we can correlate the electrical and optical signals, as illustrated in Figure 3. We investigate their fluctuations under a constant bias using a single photon counting module (SPCM) for faster acquisition synchronized with the conductance measurement. Our general finding is that light emission intensity and conductance value are strongly correlated despite the relatively large area of the junction and the presence of hundreds of molecules. It is compatible with the existence of a few molecular sites that dominate both transport and light emission and whose number fluctuates over time. For the same junction, the conductance–emission correlation can switch between positive and negative trends over extended measurement times, as illustrated in Figure 3e–g (additional data sets are provided in Supporting Information Section S9). The light emission from the IET process is, however, expected to vary linearly with current^67^ and to display abrupt changes only when switching from tunneling to quantum point contact.^24,27^ The deviation from linearity and the periods of negative correlation observed in our PMJs will now be explained by the interplay between two nanojunctions in series.

As mentioned earlier, most of the voltage drop happens across one of the two junctions in series and the light emission is expected to originate from this dominating one as illustrated in Figure 3d. Hence, the conductance *G*_1_ of the nonemitting junction is assumed to be much greater than *G*_2_ that of the light-emitting junction. Consequently, small fluctuations in *G*_1_ have insignificant impacts on overall conductance and emission intensity. The instantaneous light emission intensity depends on three major components: (i) the current through the emitting junction *i*(*t*);^27^ (ii) the voltage across the emitting junction *V*_mid_ – *V*_R_ (through the energy-dependent responsivity of the detector); and (iii) the efficiency for converting electrical energy into photons, which varies with the emitter location^42^ (see Figure 4a,b). We will show that contributions (i) and (ii) can together explain both negative and positive correlations of photon counts vs conductance observed in the same junction at different times, while (iii) results in a variation of the overall efficiency of the same PMJ accounted for by the prefactor *A* below. The intensity of light emission is therefore modeled by the equation
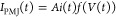
1where *f*(*V*(*t*)) is an experimentally determined function that accounts for the time-averaged voltage-dependent emission and detection efficiency. The linear dependence on *i*(*t*) is to be interpreted as a first-order Taylor expansion valid for a limited range of conductance fluctuations. From the conductance data collected at constant bias, we fit the data from Figure 3 to obtain the values of the parameters *G*_1_ and *G*_2_(*t*). These parameters are used to derive the values of *V*_mid_ – *V*_R_ for each value of the conductance.

The empirical relation from eq 1 is then used to obtain the fit shown as solid lines in Figure 3e,g. When the change in *V*_mid_ – *V*_R_ is negligible, the monotonous dependence of photoemission on the current dominates and leads to positive correlations between photon count rate and conductance. This can happen when *G*_1_ ≫ *G*_2_ such that *V*_L_ – *V*_mid_ approaches 0. This is the case for the fit parameters *G*_1_ = 10^–2^*G*_0_ and ⟨*G*_2_⟩ = 10^–5^*G*_0_ in Figure 3e (the notation ⟨*G*_*i*_⟩ is used to represent the mean value of the conductance of junction *i* = 1, 2). Conversely, negative correlations are observed when *V*_mid_ – *V*_R_ changes significantly so that the voltage dependence of emission embodied in *f*(*V*(*t*)) dominates over the dependence on current. This is the case for fit parameters *G*_1_ = 10^–3^*G*_0_ and ⟨*G*_2_⟩ = 2 × 10^–4^*G*_0_ in Figure 3g. Depending on the values of *G*_1_ and *G*_2_(*t*), the two opposite regimes can be realized. In all fits, *G*_1_ > *G*_2_ as predicted, consistent with our initial assumption that one of the two junctions has a higher conductance compared to the other one. More details on the data fitting are provided in Supporting Information Section S8 and more examples of measured and fitted correlations are presented in Section S9.

In Figure 4, we report the fluctuations in the light emission spectrum occurring together with conductance fluctuations in the same PMJ. The light emission spectrum is dominated by two peaks at photon energies of 1.56 and 1.72 eV (as estimated by the Lorentzian fit in Figure 4b), but their respective intensities can randomly change over time. Intermittent blinking in single-molecule junctions can happen from conformational or structural changes in the molecular backbone. But such effects take place in time scales of 10^–11^ to 10^–9^ s and are averaged out in large area molecular junction.^60,68,69^ A gold–SAM–EGaIn junction with a small contact area was able to capture such intermittent blinking in light emission but found no correlation with current fluctuations.^38,39^ The BPDT molecules can display conductance switching by a change in the tilt of the molecule in the junction, its binding conformation with the gold atoms, and the rotation between the two benzene molecules.^55,59^ These effects could explain a change in the intensity of the light emission resulting from a change in conductance^70^ but do not explain the reshaping of the emission spectrum.

Instead, we argue that dynamically occurring, localized conduction channels naturally explain the observed fluctuations in the light emission spectrum. We hypothesize that the changing ratio between emission intensities at the different plasmonic peaks is caused by the random appearance of point-like emitters at different positions inside the host nanocavity. Depending on its localization, a point-like emitter couples more efficiently to a particular mode of the plasmonic response. The far-field spectra of these point-like emitters depend on the overlap between the emitter position and the near-field distribution of the different gap modes. Hence, the spatial wandering of point-like emitters in the near-field causes an apparent spectral wandering in the far-field. This has been observed in photoexcited luminescence of gold clusters in a plasmonic cavity by Chen et al. in ref (42) where simulation of this effect in a gap nanocavity similar to the ones studied here was performed.

The relative conductance fluctuations in our PMJs are found to be significantly enhanced under increasing electric bias (see Supporting Information Section S7), while there is no clear temperature rise seen in the overbias emission, within the fitting uncertainty (Figure S12). These observations suggest that a nonthermal mechanism may contribute to the creation of new atomic protrusions and could be connected to recent findings on optically induced picocavities,^71^ where external electric fields (at the optical frequency) were argued to lower the energy barrier for the creation and relaxation of a gold adatom. However, future dedicated experiments are needed to confirm the nonthermal mechanism that may be at play in the junction.

## Conclusions

In summary, using a simple and scalable self-assembled geometry, we demonstrated how microscopic instabilities inside a plasmonic molecular nanojunction are imprinted on the electrical transport as well as the tunneling-induced light emission fluctuations. The nonmonotonous correlations between electrical conductance and IET intensity are explained by a simple phenomenological model taking into account that our devices consist of a double junction in series. The large intermittent fluctuations in conductance and IET emission are proposed to arise from the ON/OFF switching of localized conduction channels, the exact location of which governs the relative coupling of IET to distinct plasmonic modes. While this is not a deterministic process, we could repeatedly drive our system in this regime by applying a sufficient voltage across the junction.

With controlled capillary assembly,^72^ atomic force microscope,^26^ transfer printing^73^ or dielectrophoresis,^74^ it is possible to make on-demand single nanoparticle junctions (some preliminary results are shown in Section S11 of Supporting Information). This would enable simultaneous SERS or luminescence and electrical measurements on the PMJ, which are currently hindered by the presence of multiple nanoparticles nearby. The resulting single-nanoparticle PMJ offers a unique opportunity to connect the microscopic origins of various phenomena such as picocavity in SERS,^40^ flares in electronic Raman scattering,^43^ blinking of gold photoluminescence,^42^ and fluctuations in IET and conductance studied here. The PMJs should be particularly useful in understanding their formation mechanisms, including their dependence on the electric field^71^ and their nonthermal origin.

## Supporting Information Available

Experimental methods, intermittent blinking in conductance, native ligands vs BPDT, plasmonically enhanced emission, thermally assisted emission, stability of the devices, electrically induced fluctuations, details of the fit, additional examples, picocavity events in SERS and conductance, outlook: formation of single nanoparticle junction (PDF)
