# Supplementary material for: Light Emission and Conductance Fluctuations in Electrically Driven and Plasmonically Enhanced Molecular Junctions
Source: ACS Photonics. 2024 Jun 6;11(6):2388–96. doi: 10.1021/acsphotonics.4c00291 (PMC11191743; doi:10.1021/acsphotonics.4c00291)
Supplement: Supplementary file 1 — ph4c00291_si_001.pdf [file ph4c00291_si_001.pdf]

Supporting Information: Light Emission and Conductance Fluctuations in Electrically Driven and Plasmonically Enhanced Molecular Junctions

Sakthi Priya Amirtharaj,[†] Zhiyuan Xie,[†] Josephine Si Yu See,[†] Gabriele Rolleri,[†] Wen Chen,[†] Konstantin Malchow,[†] Alexandre Bouhelier,[‡] Emanuel Lörtscher,[¶] and Christophe Galland[*,†]

†*Institute of Physics, Ecole Polytechnique Fédérale de Lausanne (EPFL), CH-1015 Lausanne, Switzerland*

‡*Laboratoire Interdisciplinaire Carnot de Bourgogne CNRS UMR 6303, Université de Bourgogne, 21000 Dijon, France*

¶*IBM Research Europe - Zurich, Säumerstrasse 4, CH-8803 Rüschlikon , Switzerland*

E-mail: chris.galland@epfl.ch

S1 Experimental methods

S1.1 Sample fabrication

The sample fabrication procedure is illustrated in Fig. S1. A 4-inch diameter silicon wafer with a thickness of 380 μm and double-sided 300 nm SiO2 layers (NOVA wafers) is used as the substrate for the fabrication process. The substrate underwent a spin coating process, with a 700 nm layer of LOR 5A followed by a 1600 nm layer of AZ1512 photoresist.

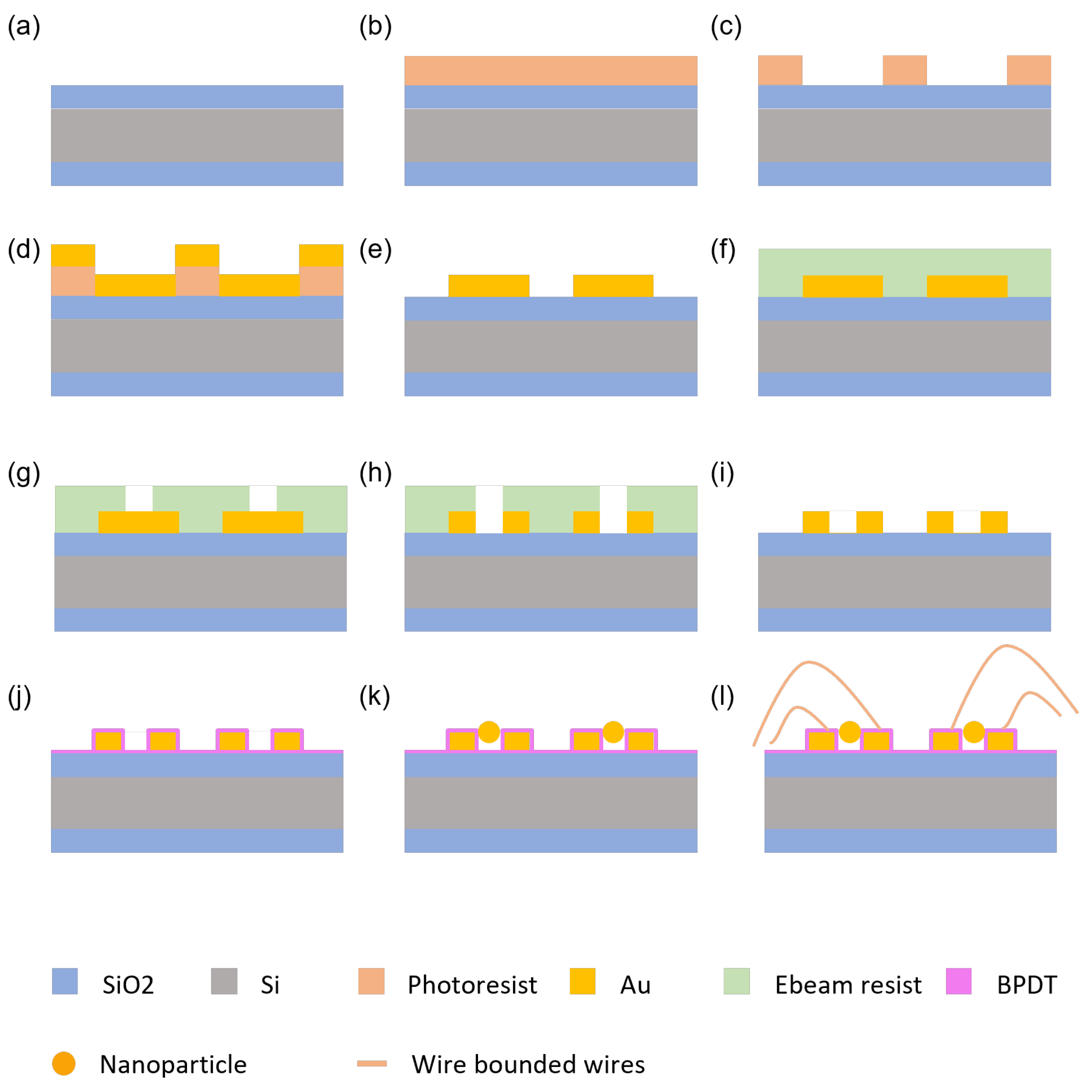

Figure S1: Fabrication steps: (a) Substrate with 380 μm Si and double-sided 300 nm SiO2 layers. (b) Spin coating with 700 nm LOR 5A and 1600 nm of AZ1512. (c) Deep-UV (DUV) photolithography. (d) Thermal evaporation of a 3 nm Cr adhesion layer followed by 150 nm of Au. (e) Lift-off process. (f) Spin coating with E-beam resist 950K. (g) Electron-beam lithography. (h) Ion-beam etching. (i) Removal of E-beam resist. (j) Formation of molecular spacer. (k) Nanoparticle drop casting. (l) Wire bonding.

A first photo-lithography is employed to define the contacts and gold electrodes by metal deposition and lift-off. Utilizing the deep-UV photolithography technique, the electrode pattern is formed on the substrate. Following the photoresist development step, a 3-nm-thick Cr adhesion layer and a 150-nm-thick gold layer are thermally evaporated onto the substrate at a rate of 0.5 nm/s. The subsequent metal lift-off process is performed using Remover 1165 - NMP.

Subsequently, electron-beam lithography and collimated ion-beam etching are employed to carve the $\sim$ 150 nm gaps separating the two electrodes, creating the space for the bridging gold nanoparticles to

fit. We used 100 keV electron-beam lithography and PMMA 950 K as the e-beam resist. Following the development of the resist, the nanogaps are etched with a collimated ion-beam at an angle of -10°, resulting in a 150 nm wide and 4 μm long V-shape trenches, oriented perpendicular to each electrode. The actual length of the gold electrode is 2 μm; the e-beam step creates a longer gap that extends to the substrate to safely mitigate alignment errors. To remove PMMA, the sample is immersed in warm Remover 1165 - NMP for a duration of 6 hours.

After the fabrication of electrodes, the wafer is diced into individual chips, with each chip size (7 mm x 10 mm) containing 25 pairs of electrodes. Following the dicing process, the chips underwent the formation of molecular spacer and nanoparticle bridges, as described in section S1.2. Chips are then forwarded for wire bonding to connect the electrodes to a printed circuit board (PCB). This wire-bonding step enabled the electrical connection between the fabricated structures and the external circuitry, facilitating further characterization with the setup described in section S1.3.

S1.2 Formation of molecular spacer and nanoparticle bridge

Biphenyl-4,4'-dithiol (BPDT) molecules in solid form from Sigma Aldrich are dissolved in ethanol to form a 3 mM solution. Each chip is incubated in 3 ml of this solution for 2 hours.[S1] The sample with the self-assembled monolayer (SAM) of BPDT is then cleaned several times with ethanol to remove unbound molecules. To form the nanoparticle bridge, 5 μl of 1:100 diluted *Nanopartz* 150 nm OD100 gold nanoparticle solution is drop cast on the sample and evaporated until the edge of the droplet passes across the slit. Due to the capillary forces, the edge has a higher concentration of nanoparticles and leaves some nanoparticles in the slit.[S2] In the final device, the electrodes with molecular SAM are bridged by a few nanoparticles as shown in the SEM image in Fig. S2.

We found that instances with a single nanoparticle have a low probability of establishing good contact with the electrodes. Having more nanoparticles increases the probability of making a successful contact. Note that all the discussion in the main text does not rely on having a single particle bridging the electrodes; having more than one simply increases the total number of molecules potentially participating in conductance and light emission by inelastic electron tunneling. Similarly, the different plasmonic modes that enhance and reshape the emission spectrum may equally be associated with distinct nanoparticles, our model remains fully valid.

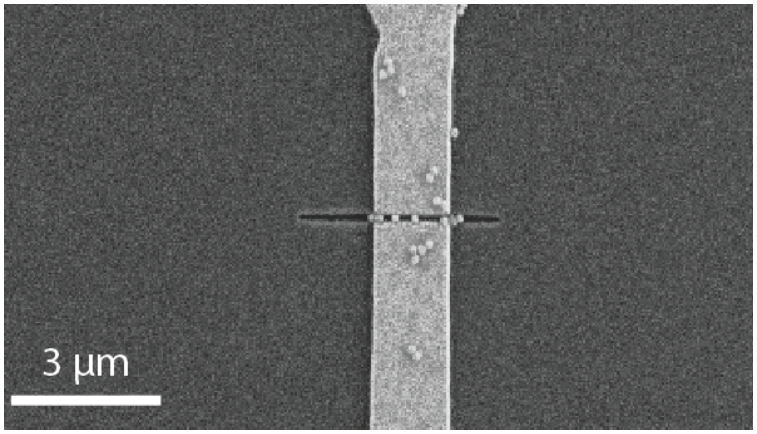

Figure S2: SEM image of a plasmonic molecular junction (PMJ) with multiple nanoparticles in the gap.

S1.3 Experimental setup

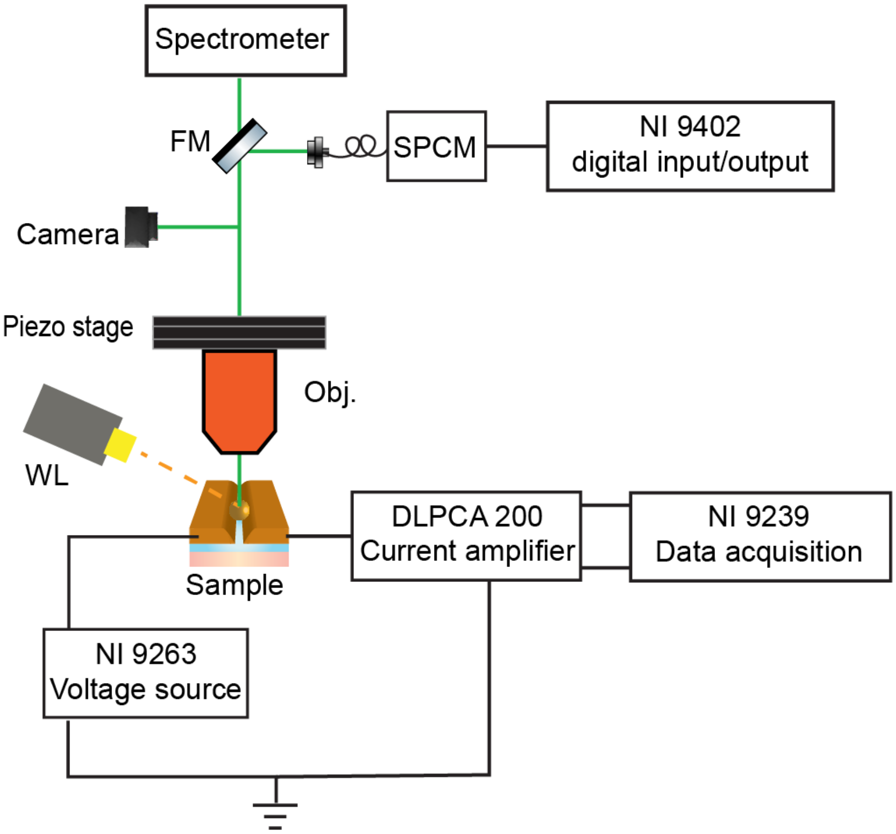

Figure S3: Schematics of the experimental setup: FM- Flip mirror; WL- White light source; Obj.- 0.8NA Objective.

The samples are investigated with a home-built optical setup shown in Fig. S3. The optical signal from the sample is collected using a 0.8 NA objective with 100X magnification and directed to an Andor spectrometer or fiber-coupled to a single photon counting module (SPCM) depending on the measurement. All results discussed in the main text are not normalized to the detection efficiency of the setup. A white light source can illuminate the sample along the laser path or from the side to obtain bright field or dark field imaging, respectively.

To perform electrical measurements, the electrodes are wire-bonded to a PCB (Fig. S4) that is then connected by wires to an external circuit consisting of a voltage source and current measurement unit. The NI 9263 voltage output module can output between -10 V and +10 V. Current-voltage characteristics are measured by applying a triangular voltage sweep. The current is fed to the DLPCA 200 transimpedance amplifier which is automatically gain-switched depending on the magnitude of the measured current by a home-built LABVIEW program. The voltage output of the current amplifier is then read by the NI 9239 analog input device. All the devices are connected to a NI 9040-cRIO controller and are synchronously operated via LABVIEW with an internal clock. Finally, to synchronize the optical measurements with the spectrometer, the NI 9402 pulse generator is used to send a trigger pulse to start the spectral acquisition. The NI 9402 also acts as a digital readout module for photon counting measurements with SPCM.

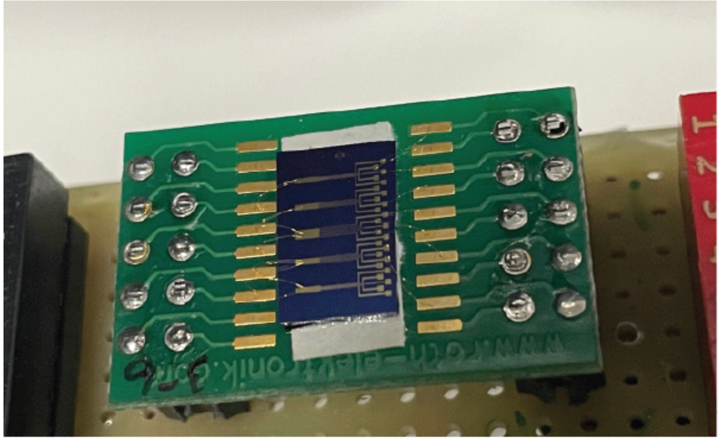

Figure S4: Image of a chip with electrodes wire-bonded to a PCB, which will be further connected to an external electrical circuit.

S2 Intermittent blinking in conductance

Intermittent blinking is frequently observed in the conductance measurements of the PMJs. A histogram of conductance values indicates two distinct peaks, as shown in Fig. S5 for repeated measurements across 6 different PMJs under a D.C. voltage of 5 mV.

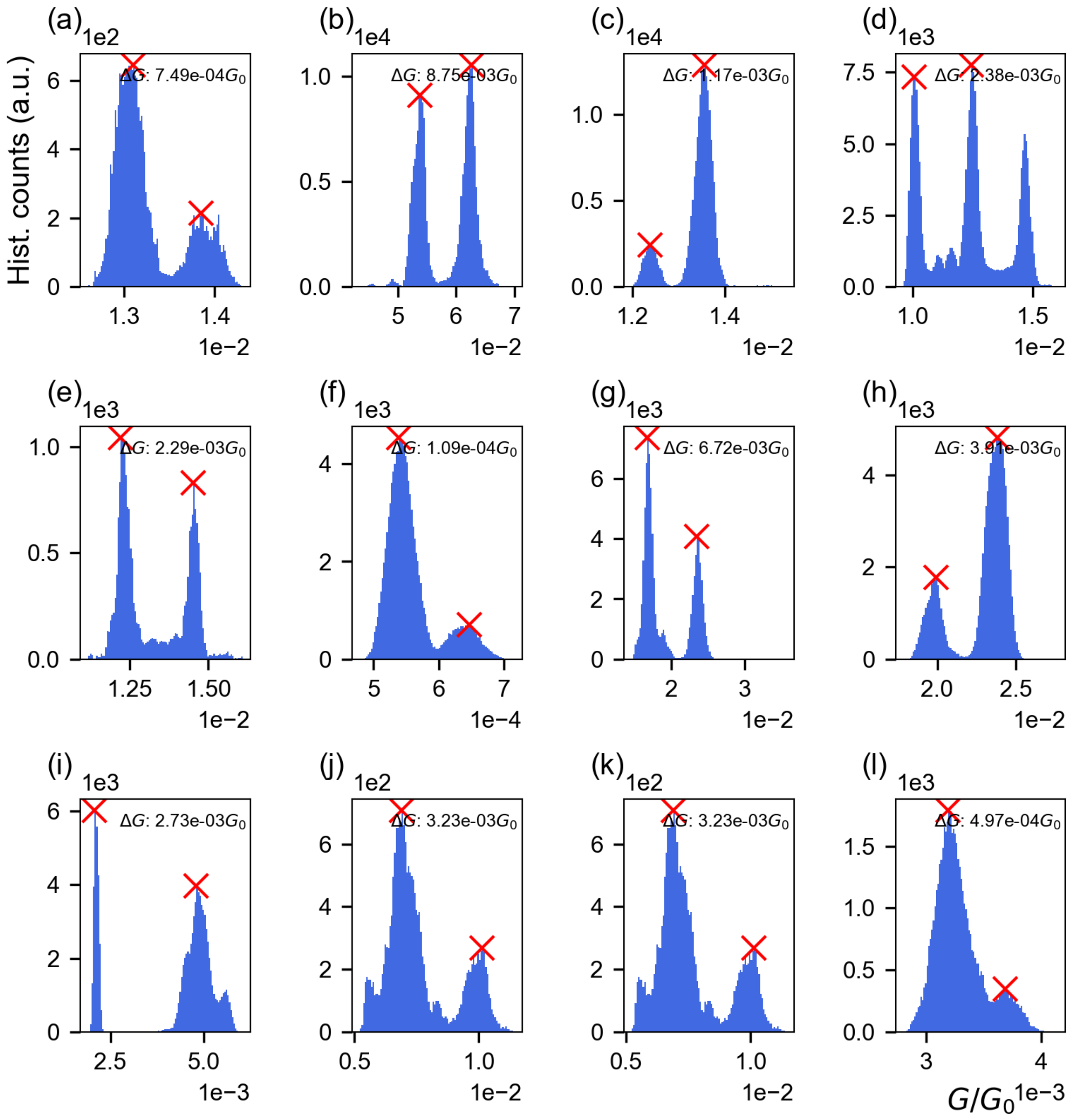

Figure S5: Intermittent blinking in conductance observed in different measurements across different devices at a D.C. voltage of 5 mV

The magnitude of the conductance jump lies between 1×10^{-4} G_0 and 8.7×10^{-3} G_0 and is in agreement with the conductance of a single BPDT molecule found in literature.[S3–S6] The conductance change due to conformational change of the molecules is much lower in magnitude.[S7,S8] The appearance of three peaks in the histogram (Fig. S5d) that are equally spaced also indicates that it does not originate from conformational changes of the molecules.

S3 Native ligands vs. BPDT

The gold nanoparticles are non-covalently capped with citrate molecules to avoid aggregation of the suspension. When these nanoparticles are deposited in the gap between the electrodes, the BPDT molecules on the surface of the electrodes are expected to replace the citrate layer to form the molecular junction, but some ligand molecules could still be present in the nanogap. To better understand how citrates impact the behavior of the junction, we compare here BPDT junctions with pure citrate-spaced junctions (no incubation in BPDT).

S3.1 Yield of the devices

Comparing BPDT and pure citrate spacers (2×3 chips, i.e. 75 devices each), the yield of BPDT in establishing measurable electrical contact is 43% while that of citrate is 23% (Fig. S6). The dashed line marks the measurement limit of our probe station, and the devices with conductance below this range are considered open circuits. Considering only the PMJs that we identify as molecular contacts ($10^{-5}G_0 < G < 10^{-1}G_0$), the histogram peaks at around $3 \times 10^{-4}G_0$ for the BPDT junction and around $3 \times 10^{-2}G_0$ for the citrate junctions, which could be related to the smaller size and possibly flatter orientation of the citrate molecules. Here, $G_0 = 2e^2/h$ refers to the quantum of conductance.

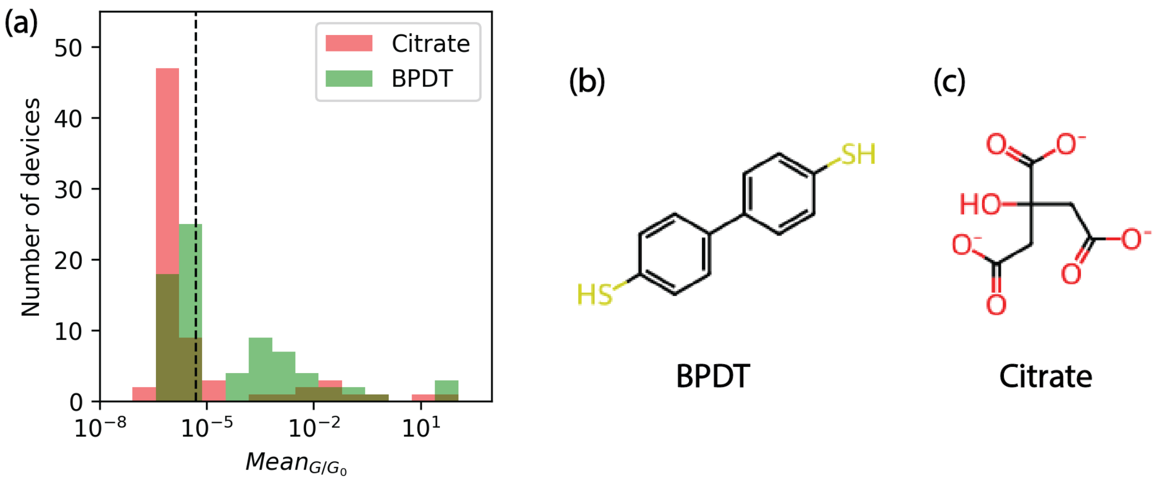

Figure S6: (a) Conductance measured at DC bias of 5 mV for several devices with BPDT (green) and citrate (red) spacer. The dashed line marks the measurement limit of the probe station. BPDT PMJs have a yield of 43% and the conductance histogram has a peak around $3 \times 10^{-4}G_0$. Citrate PMJs have a yield of 23% and the conductance histogram has a peak around $3 \times 10^{-2}G_0$. Molecular structure of (b) BPDT and (c) citrate molecule

S3.2 Current-Voltage characteristics

The current-voltage characteristics of several devices with BPDT and citrate spacer are shown in Fig. S7

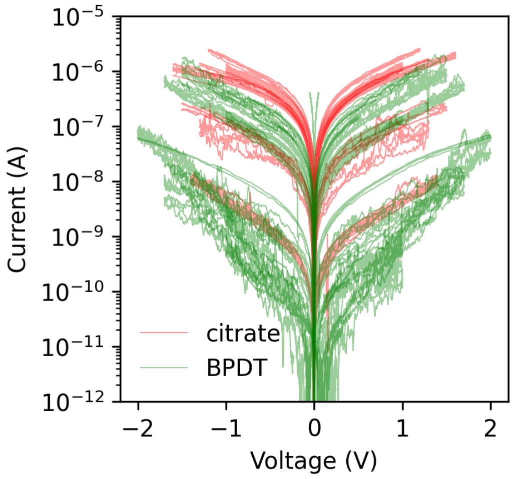

Figure S7: Current-voltage characteristics of several devices with BPDT (green) and citrate (red) spacer.

A qualitative observation is that PMJs with BPDT spacers are found to feature more pronounced fluctuations in conductance than citrate spacers. However, to clearly distinguish the two kinds of PMJs through their I-V curves, we believe that cryogenic, molecular-specific characterization like inelastic tunneling spectroscopy[S9] is required.

S3.3 Inelastic electron tunneling (IET) light emission

We observe light emission by IET from both kinds of PMJ (with citrate and BPDT spacers). Our PMJs get damaged when the current through them exceeds a few μA and hence a current compliance of 1 μA is maintained. As a consequence, PMJs with higher conductance (above $2 \times 10^{-3} G_0$ at 5 mV) cannot be used for IET experiments as their currents under the voltages where IET becomes measurable are higher than the current compliance.

The voltage threshold at which IET is detected is shown for different PMJs with citrate and BPDT spacer (Fig. S8). For most devices, the voltage threshold is close to the cut-on energy of the detector efficiency. For other devices with lower conductance, the photon yield is low and they require much higher voltages for IET to be detectable.

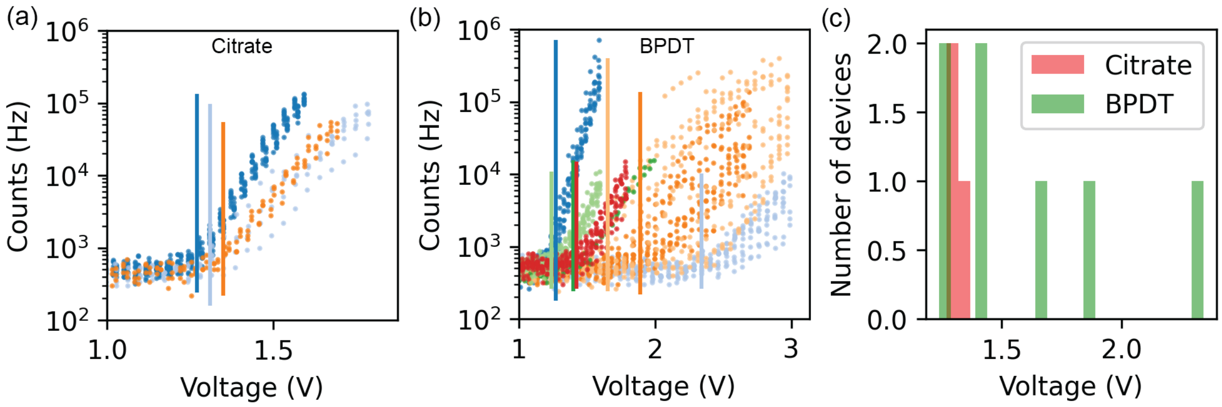

Figure S8: Photon counts versus voltage for several PMJs for (a) citrate spacer and (b) BPDT spacer. Vertical lines mark the voltage threshold for onset of observable IET. (c) Voltage threshold plotted in the form of histogram.

S4 Plasmonically enhanced emission

The design of the nanoparticle-SAM-electrode is adapted from previous work[S10] where the numercial simulation of the plasmonic modes was performed. (In Ref.[S10] the 2 µm groove acted as an antenna at mid-IR wavelengths, which is not needed here). The simulations predict at least two distinct radiative modes at visible wavelengths that arise from the hybridization of the individual gap modes formed by each nanoparticle-on-mirror (NPoM) cavity between the nanoparticle and each side of the electrode.[S11] Due to the added complexity in the electrically-contacted device studied here that often involved multiple nanoparticles in different positions within the nanogroove, we did not repeat the simulation of the plasmonic modes for the individual devices and refer the reader to Ref.[S10] for a general overview of the plasmonic response.

The electrical excitation of localized plasmons is confirmed from the dark-field scattering and the polarization response. We first obtain the dark-field scattering spectrum from the junction for P and S polarization of the incoming white light. The dark-field scattering spectroscopy is obtained using illumination from a tungsten lamp at about 15° incidence angle with respect to the sample plane as illustrated in Fig. S3. The spectrum is collected using a multimode fiber and measured with a QE pro-Ocean Insight spectrometer. The S-polarized excitation results in two dominant scattering peaks that are suppressedd under P polarization. The same peaks are excited by the IET process with an external bias voltage, indicating that they are of plasmonic origin. The dark-field scattering spectrum and IET emission of a PMJ at 1.9 V is shown in Fig. S9 for mixed polarization. Some additional features in the dark-field spectrum are not found in the IET spectra because the dark-field scattering signal comes from the entire junction in the collection area including the nanocavities formed by multiple nanoparticles in the electrode gap. IET on the other hand couples to the

plasmonic response of the conducting nanoparticle bridge only.

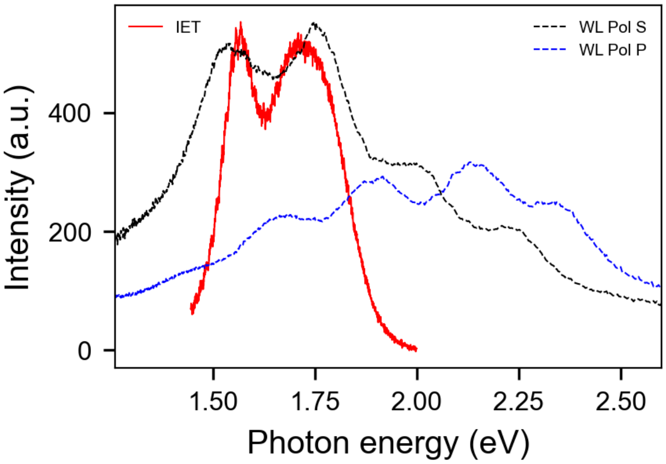

Figure S9: Light emission spectra at 1.9 V (red) and dark-field scattering spectra of a PMJ with S-(dotted black) and P-Polarized (dotted blue) excitation.

To evidence the role of plasmonic resonances in the out-coupling of light emission, a linear polarizer is placed in front of the spectrometer, and the light emission spectrum is collected along different electric field orientations (Fig. S10). The variation of the spectrum with the polarization angle is consistent with the enhancement and reshaping of emission by the plasmonic cavity.

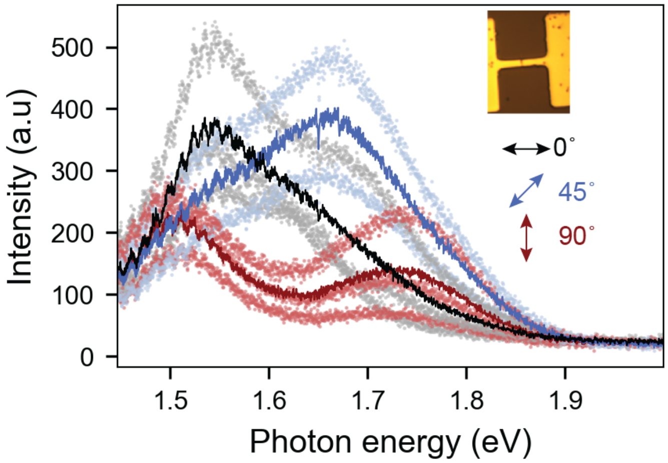

Figure S10: Light emission spectra at 1.8 V collected for various orientations of a polarizer in front of the spectrometer (0°-black; 45°-blue; 90°-red represent the angle of the polarizer with respect to the electrode length), showing spectral reshaping of emission through the plasmonic cavity. Dark lines are the averages of individual exposures shown in faded lines. Inset - bright field image of the PMJ showing the orientation of the electrode. In all experiments of the main text, no polarizer is used in the detection path.

The presence of an efficient plasmonic cavity is also evident from the observation of surface enhanced

Raman scattering by the molecular monolayer. The plasmonic resonances at visible and NIR wavelengths[S10] enable us to detect the vibrational signal from the molecules. In Fig S11, we show the SERS signal of a PMJ with BPDT molecules. When no nanoparticle is present, the Raman signal is very weak. The presence of nanoparticles increases the electromagnetic field enhancement and also enhances emission from the molecular vibration, hence enhancing the SERS signal.

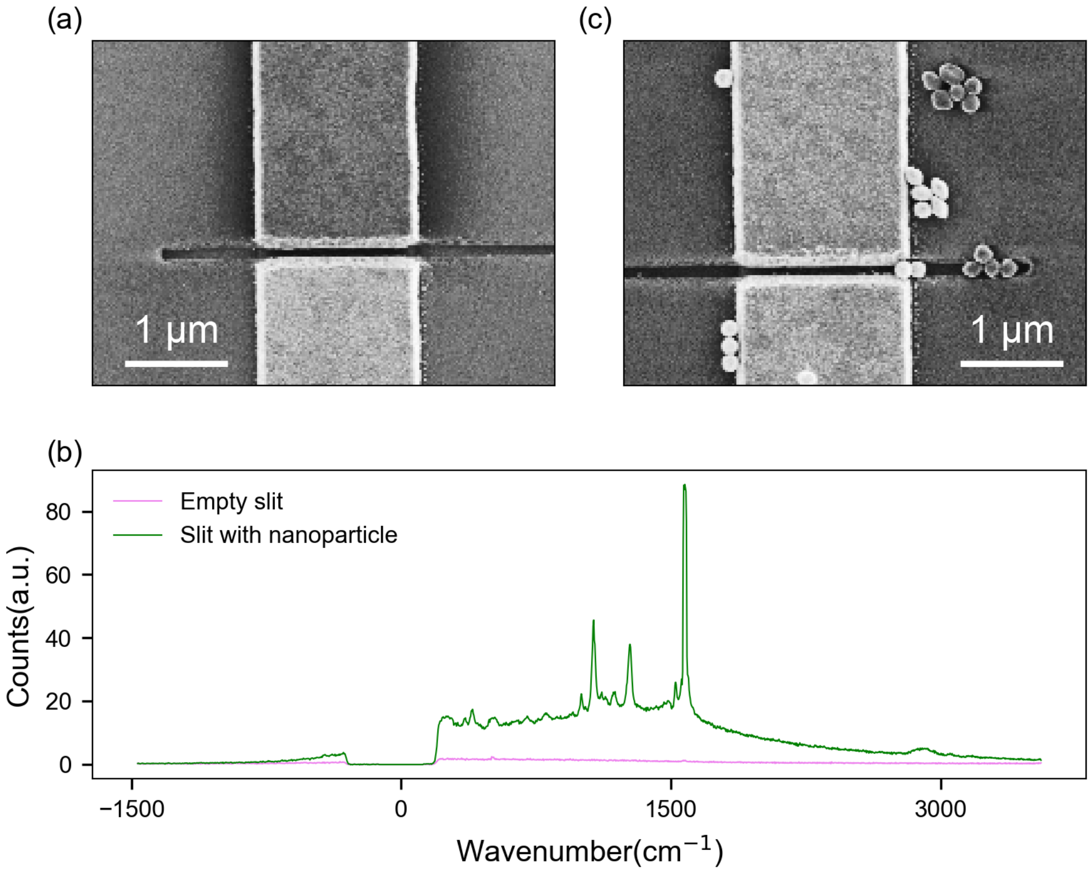

Figure S11: SEM image a PMJ with (a) electrode alone and (b) electrode with nanoparticle in the gap. (c) Their corresponding SERS spectra.

S5 Thermally assisted emission

The overbias contribution to the light emission spectra is attributed to the emission from a globally hot system at equilibrium. While it is possible for the electron temperature to be significantly higher than the lattice temperature, the observed overbias tails do not require such an assumption to be explained in our experiment. Fig. S12 shows the temperature estimated from the tail of the overbias emission with Boltzmann

fit,

$$I_{thermal}(\lambda) = \frac{A.hc}{\lambda.(exp(\frac{hc}{\lambda k_B T}) - 1)} + B \quad (1)$$

where λ is the wavelength, h is Planck's constant, c is the speed of light, k_B is Boltzmann constant, T is temperature, A and B are constants. Even without factoring out the plasmonic response (which is difficult to do properly due its fluctuating and voltage-dependent contribution) the thermal fit reasonably estimates slightly elevated temperatures of the PMJ compared to room temperature.

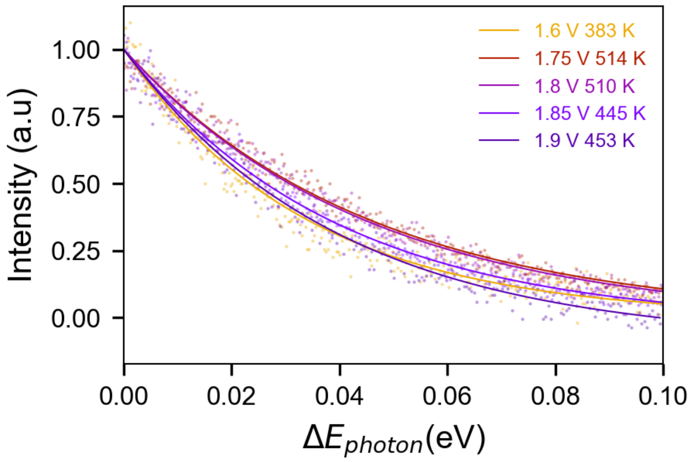

Figure S12: Thermal fit of the light emission spectra in the overbias region for various DC bias voltages (exposure time - 20 s). The energy values in the x-axis are computed with respect to the maximum photon energy corresponding to the bias voltage.

S6 Stability of the devices

The PMJ devices are working for several days and their conductances monitored over time in between measurements are shown in Fig. S13. The conductance state changes over time mostly *during measurements* as shown for two devices in Fig. S13a-b. These two PMJs, for example, remained functional for more than 100 days.

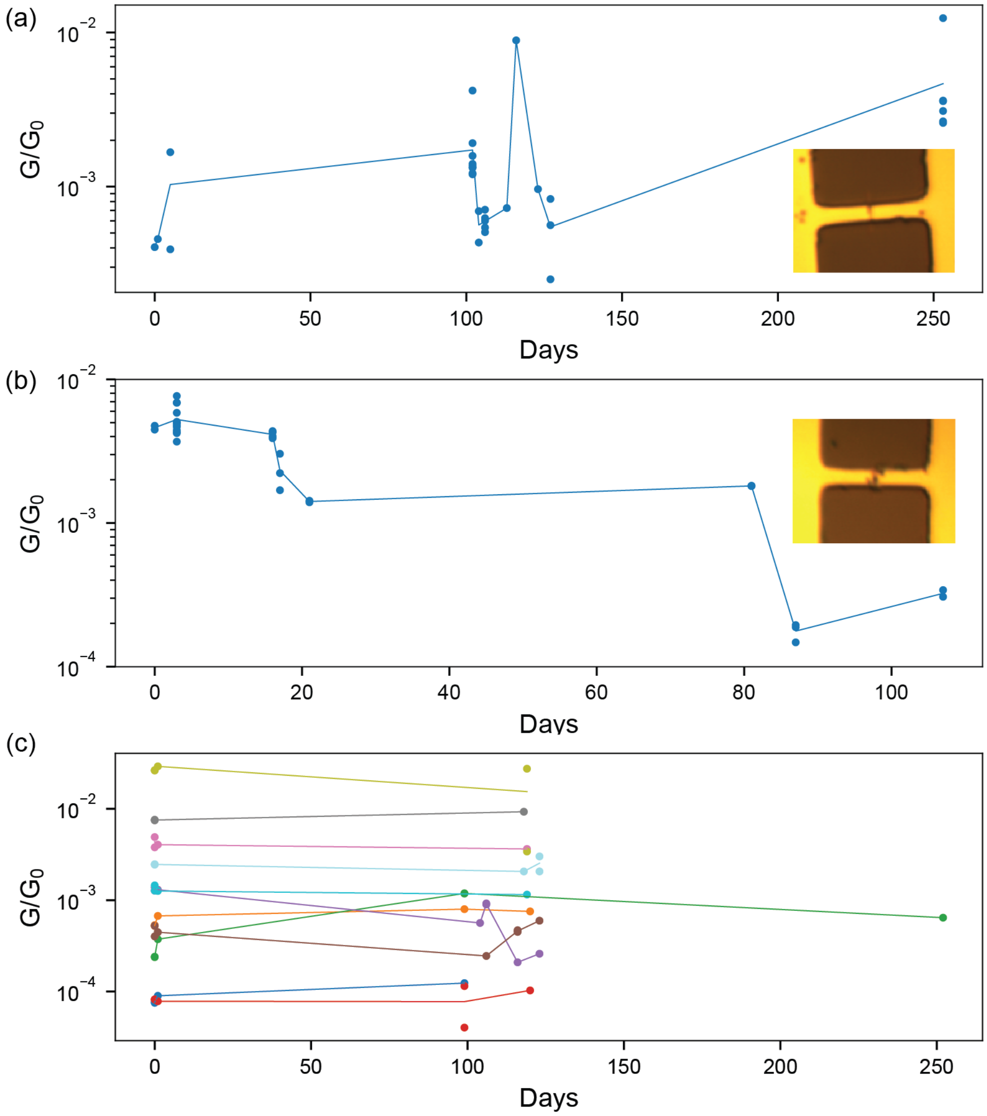

Figure S13: Conductance of two repeatedly measured PMJs (a)-(b) and several other PMJs (c) over several days (DC bias - 5 mV). Each color in (c) corresponds to an individual PMJ. The devices show excellent long-term survival.

A time trace of continuous conductance measurement for 1 hour is shown in Fig. S14

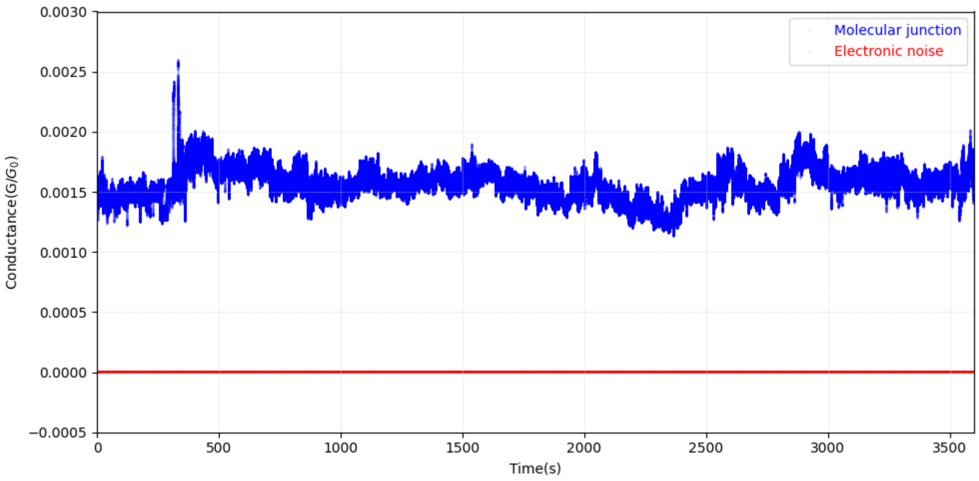

Figure S14: Stability of a PMJ for 1 hour (DC bias - 5 mV) in blue. Red line shows the open circuit noise from the electronic measurement unit.

S7 Electrically induced fluctuations

The random fluctuations in the conductance are found to intensify with an increase in the applied bias voltages, as illustrated in Fig. S15. The standard deviation of the conductance from each measurement is monotonously increasing with the increase in the voltage both in the forward and reverse sweep of voltages (Fig. S15c). The device is prone to significant changes in its conductance state at high voltages as observed in the transition of conductance at 1.5 V. This indicates that fluctuations are strongly current- or voltage-driven, possibly involving a non-thermal mechanism given the very good heat dissipation provided by the large gold electrodes.

Yet, we remark that once the standard deviation of the conductance is further normalized by its mean value (Fig. S15d), the hysteresis from Fig. S15c disappears. Two lines of explanation are proposed. First, it could be that more fluctuations correlate with a higher number of conducting channels. Second, it may indicate that more fluctuations correlate instead with the increase in the dissipated power through local Joule heating. Distinguishing the thermal and non-thermal contributions to the conductance fluctuations therefore requires further investigation of these effects with spectroscopic thermometry techniques or direct temperature scanning experiments.

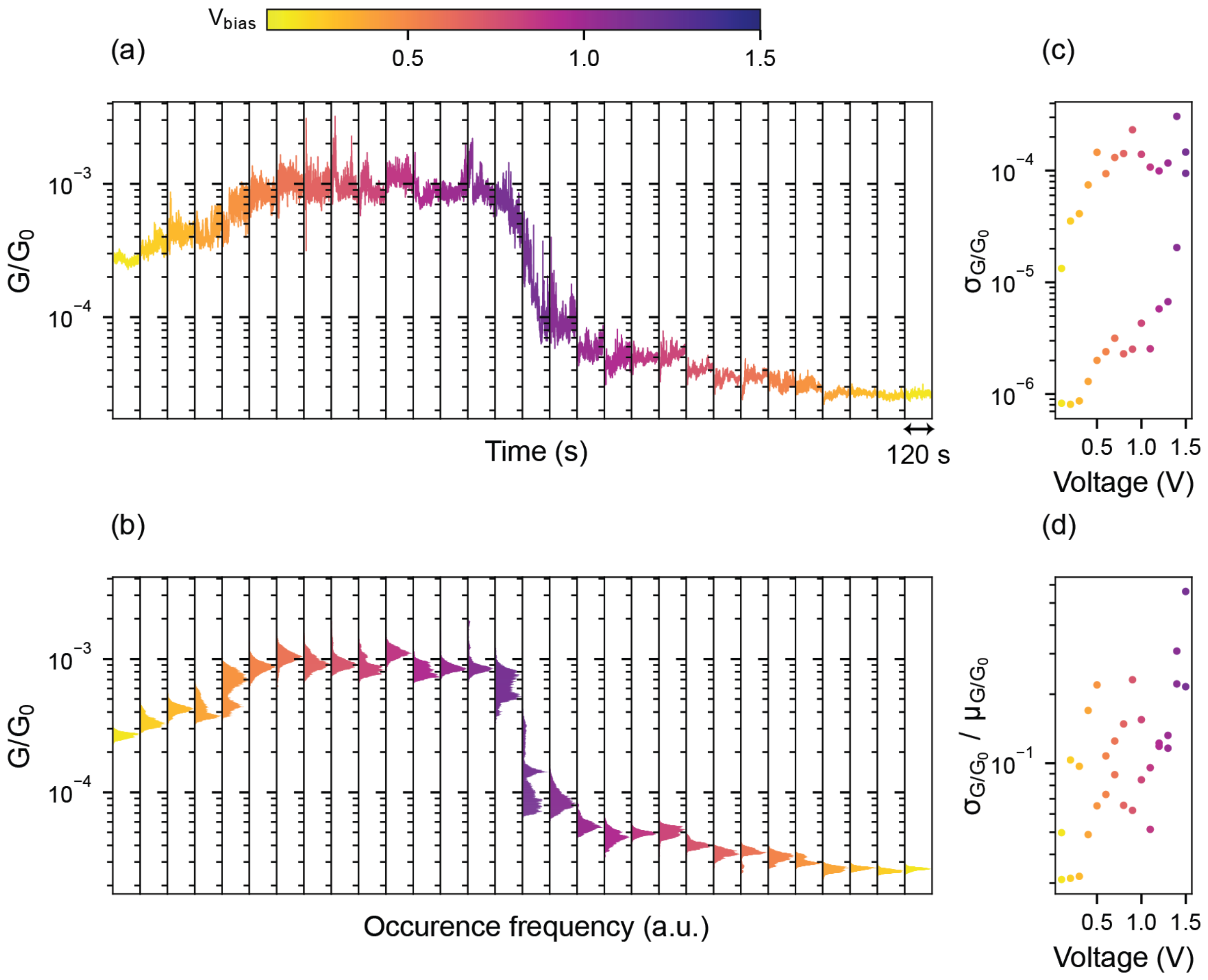

Figure S15: (a) Conductance of a BPDT junction measured for 120 s consecutively at each voltage from 0.1 V to 1.5 V and back to 0.1 V in steps of 100 mV. (b) Corresponding histograms of the conductance data. (c) Standard deviation (σ) of the conductance of each measurement in (a) plotted against the applied DC voltage. (d) The standard deviation of the conductance divided by its mean value (μ) plotted against the voltage.

S8 Details of the fit

To determine the photon emission as a function of conductance for a fluctuating PMJ, we model the intensity of light emission by the equation

$$I_{PMJ}(t) = A\,.i(t)\,.f(V(t)) \tag{2}$$

where A is a scaling factor to account for the overall efficiency of IET and photon detection, $i(t)$ is the current, and $f(V(t))$ is an experimentally determined function that accounts for the voltage-dependent photo-detection efficiency.

As most of the voltage drop happens across one of the two junctions in series, the light emission is expected to originate from this dominating one. Hence, the conductance G_1 of the non-emitting junction is assumed to be much greater than G_2, the light emitting junction. Consequently, small fluctuations in G_1 have insignificant impacts on overall conductance and emission intensity. If $i(t)$ is the current flowing across this junction when the conductance G_2 is allowed to vary with time, then

$$V_{mid} - V_R = \frac{i(t)}{G_2(t)} \tag{3}$$

$$i(t) = V_{bias} \cdot \frac{G_1 . G_2(t)}{G_1 + G_2(t)} \tag{4}$$

where $V_{bias} = V_L - V_R$.

From the measured current values under constant DC bias, we obtain the values of the fit parameters G_1 and $G_2(t)$ in Equation. 4. These parameters are used to derive the values of $V_{mid} - V_R$ for each value of the conductance, which in turn is used to estimate the values of $V_L - V_R$ as

$$V_L - V_R = \frac{G_1 + \langle G_2(t) \rangle}{G_1} . (V_{mid} - V_R) \tag{5}$$

The voltage fluctuation $V(t) = V_L - V_R$ is then known. To estimate the photon counts from the voltage, we need to determine the function $f(V(t))$ in equation 2. To obtain this, we measure the photon emission as a function of applied bias voltage (Fig. S16) and fit the curve with an equation,

$$f(V) = \begin{cases} C + B & \text{if } 0 \leq V \leq V_0 \\ C \cdot e^{k \cdot (V - V_0)} + B & \text{if } V > V_0 \end{cases}$$

where C, B, k and V_0 are fit parameters for the piecewise exponential function.

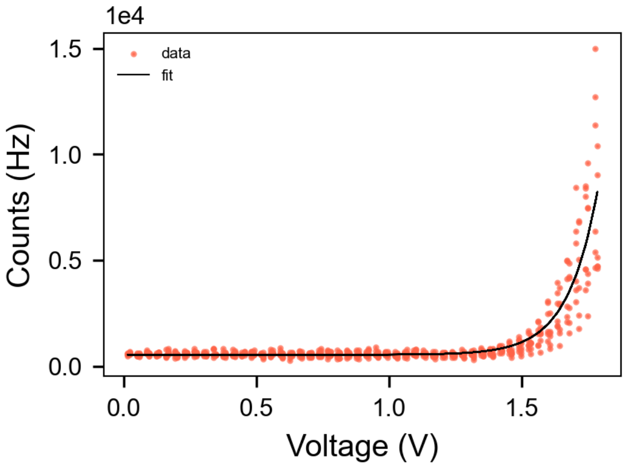

Figure S16: Experimental data of photon emission as a function of voltage (red dots) and the corresponding fit (black line).

Finally, from the values of $f(V(t))$ and $i(t)$, the emission from PMJ can be fit with the equation 2 with a factor A. The fit obtained for different sets of the fitting parameters is shown in Fig. S17.

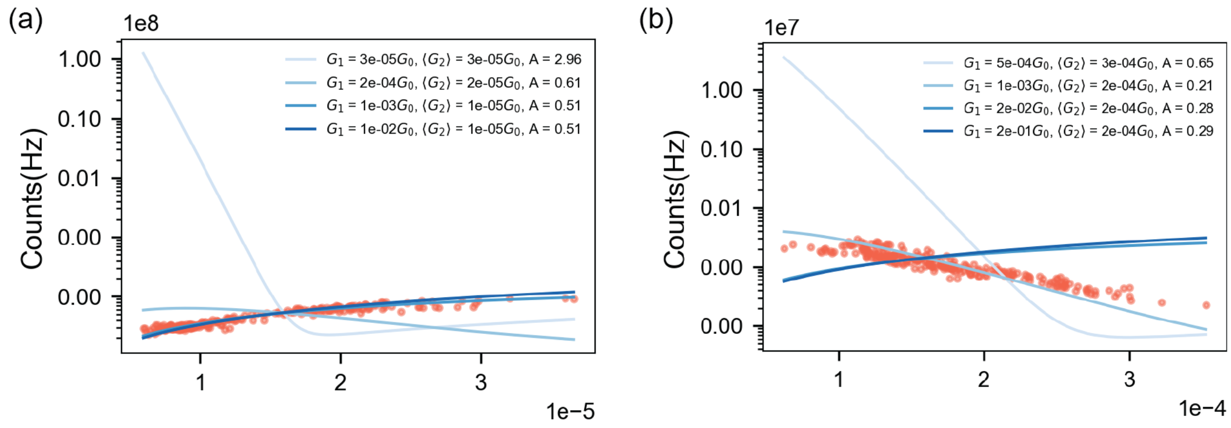

Figure S17: Multiple fits of experimental data discussed in the main text with different values for G_1, $G_2(t)$, and A for (a) positively correlated and (b) inversely correlated regimes of conductance.

S9 Additional examples

S9.1 Light emission from PMJ with varied number of nanoparticles

Correlatated fluctuations in conductance and light emission obtained from different PMJs with BPDT spacer and varied number of nanoparticles in the electrode gap are shown in Fig. S18.

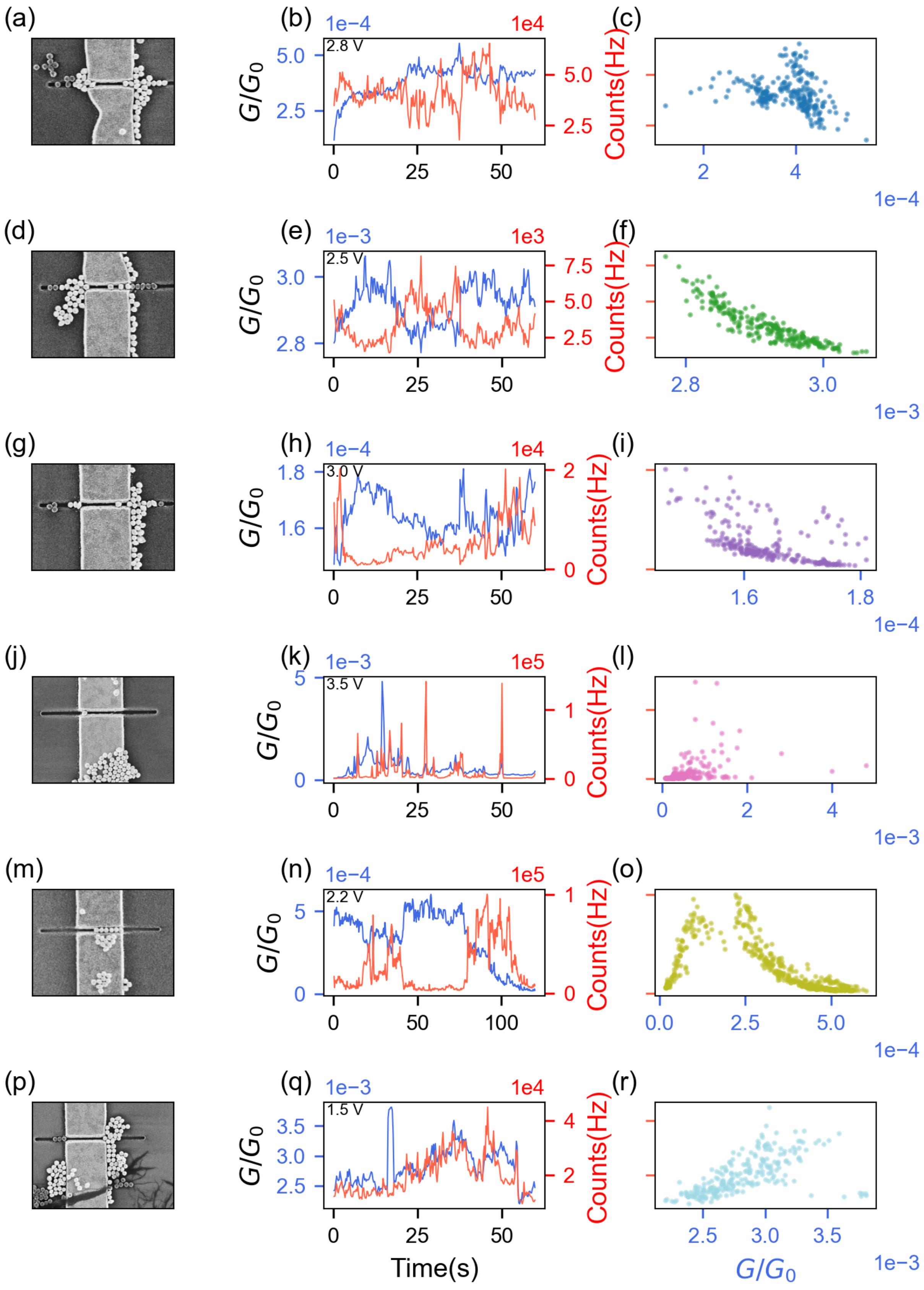

Figure S18: Correlatated fluctuations in conductance and light emission obtained from different PMJs with varied numbers of nanoparticles in the electrode gap along with their SEM image

Note that the measurements are performed at different D.C. voltages for each device to collect good signal from light emission. Each device could have a slightly varied threshold for light emission as discussed in Sec. S3.3. The summary of fluctuations in conductance and light emission from these devices is shown in Fig. S19. The conductance and light emission do not show any absolute scaling with the number of nanoparticles. Thus we conclude that despite the presence of multiple nanoparticles, only very few nanoparticles make successful electrical contact. Each device is slightly different in terms of conductance and light emission.

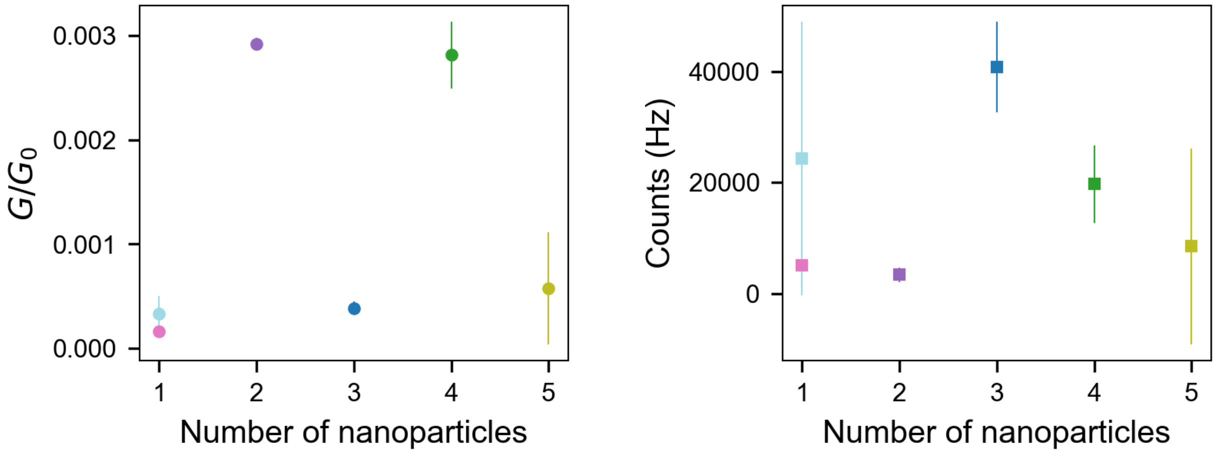

Figure S19: (a) Conductance and (b) Photon counts represented with errorbar for the devices in Fig. S18, with the color of each data point corresponds to the color of the correlation scatter plot.

S9.2 PMJ with BPDT spacer

Another example of a PMJ showing the two regimes of conductance correlations is shown in Fig. S20 for a BPDT spacer. Noteworthy is the stability of conductance and emission intensity values observed between the end of a measurement and the start of the next one. This observation further suggests that the fluctuations are current- or voltage-driven, with little contribution from ambient thermal energy.

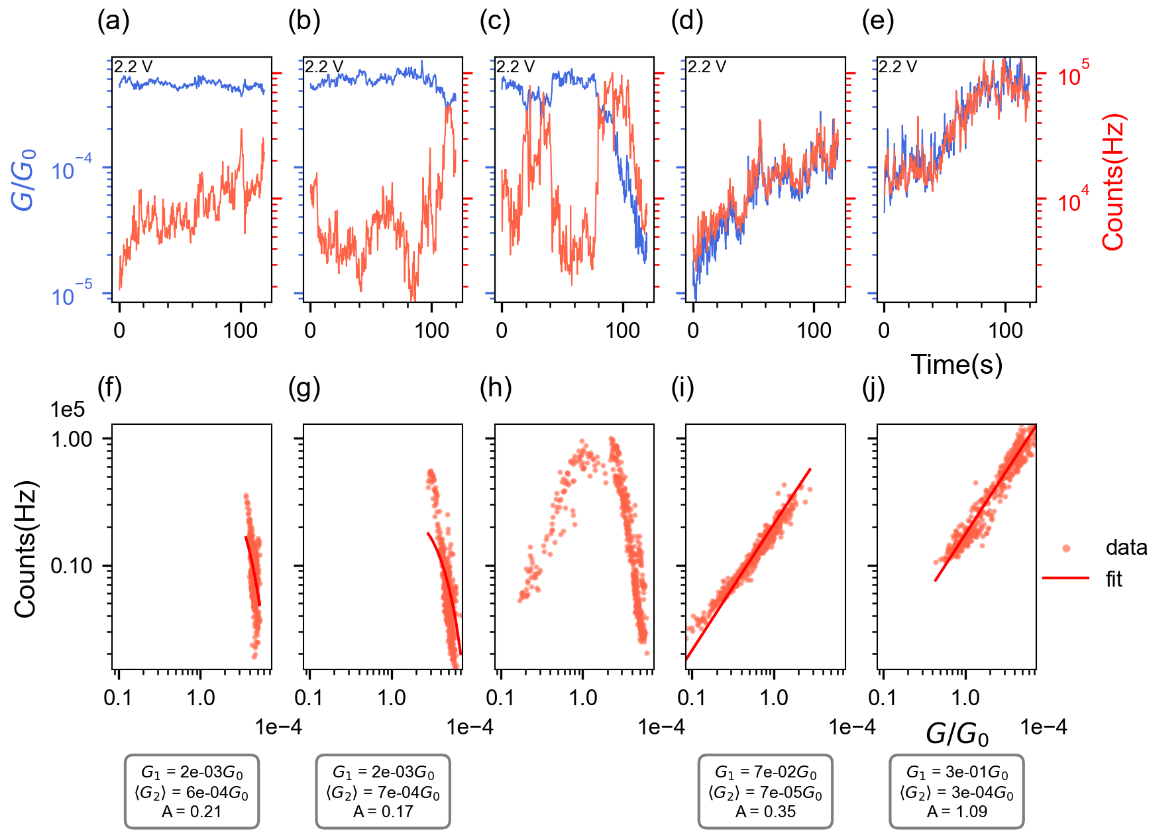

Figure S20: (a)-(e) Conductance (blue lines) and photon counts (red lines) simultaneously measured with SPCM for a particular PMJ with BPDT spacer. Both data sets are summed into 500 ms time bins. (f)-(j) corresponding correlation plots displaying the switching between positive and negative correlations. Solid line in each plot depicts the fit of the experimental data and the corresponding fit parameters are mentioned in the box below.

More measurements performed at different DC bias voltages on a few other devices showing a monotonous (either positive or negative) conductance-emission correlation are shown as a summary in Fig. S21

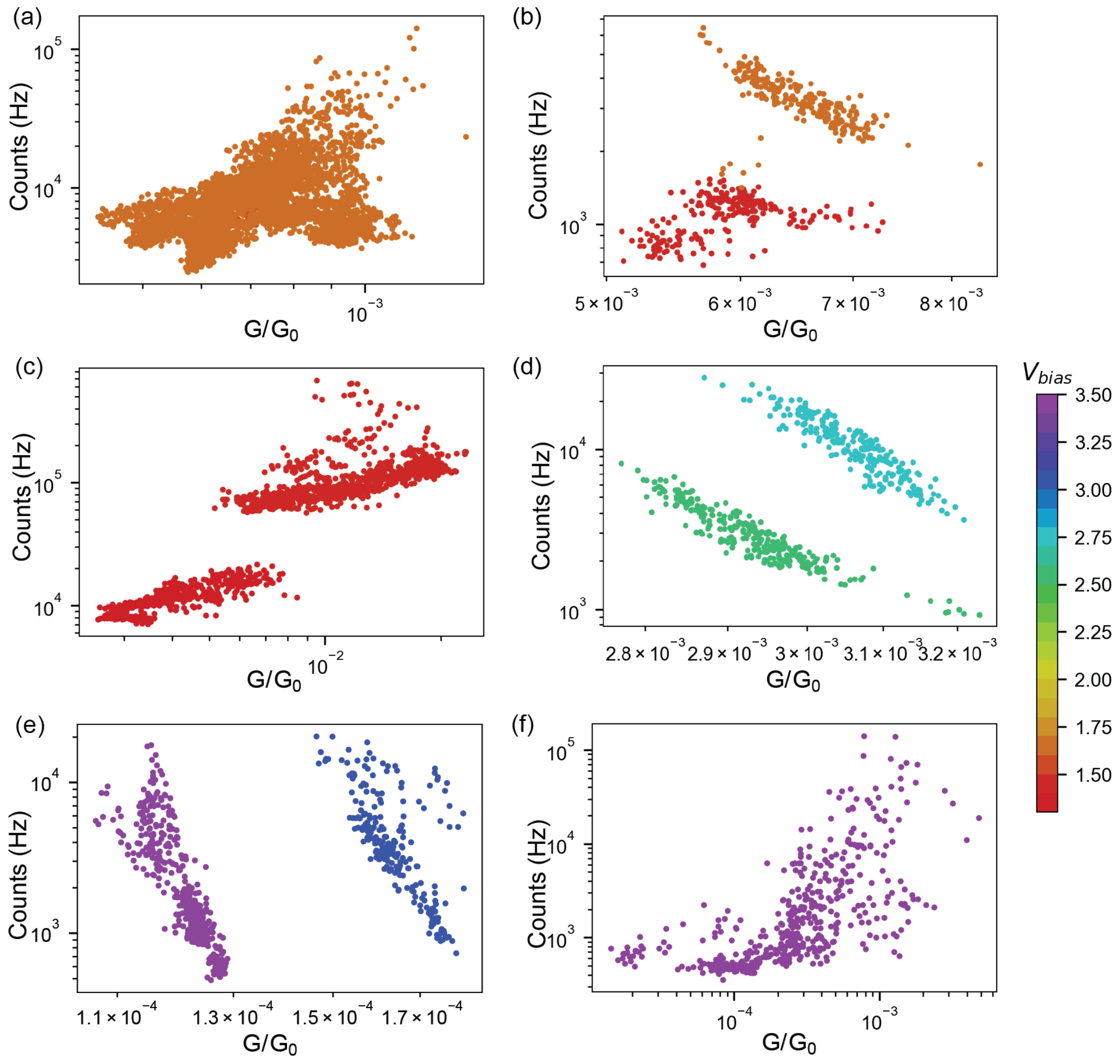

Figure S21: Correlation between photon count rate and conductance measured on different BPDT-spaced PMJs. Each plot from (a)-(f) represents an individual PMJ. The different colors indicate the corresponding applied DC bias.

S9.3 PMJ with citrate spacer

Some additional examples of PMJs with citrate spacer are shown in Fig. S22 and Fig. S23.

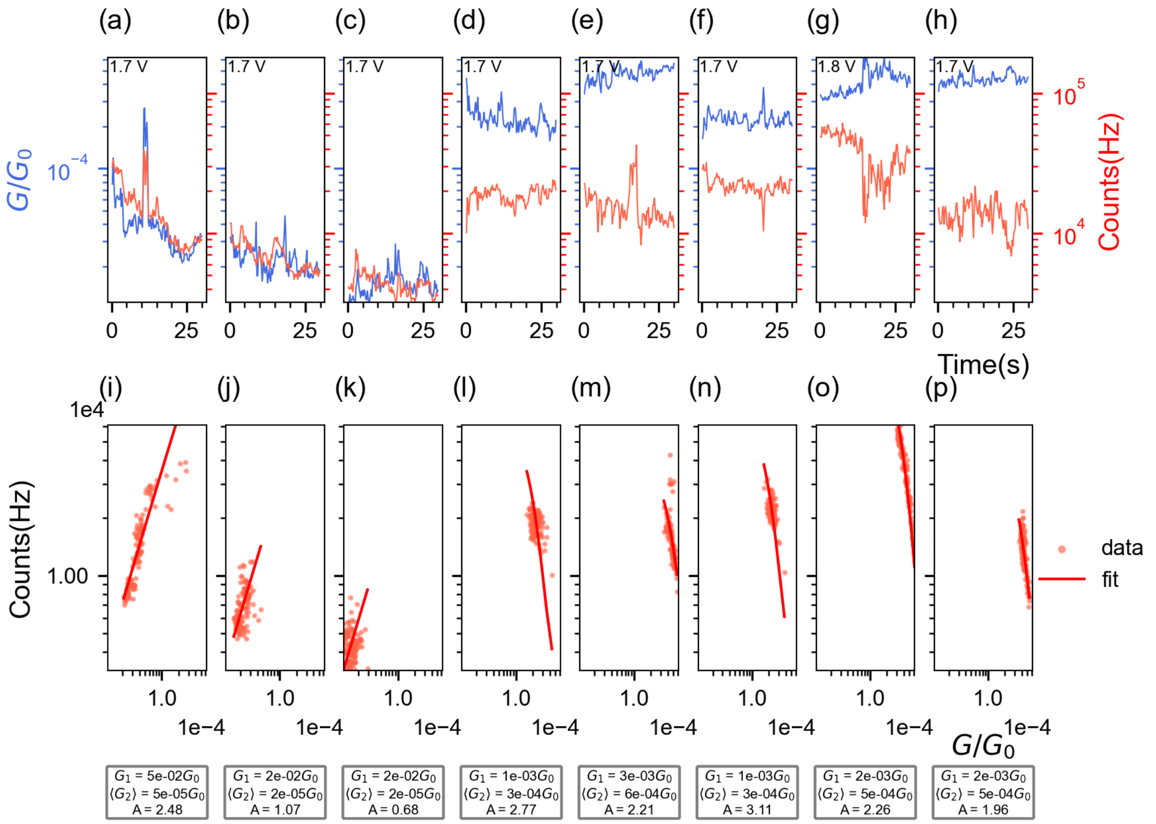

Figure S22: (a)-(h) Conductance (blue lines) and photon counts (red lines) simultaneously measured with SPCM for a particular PMJ with citrate spacer. Both data are summed into 500 ms time bins. (i)-(p) corresponding correlation plots displaying the switching between positive and negative correlations. Solid line in each plot depicts the fit of the experimental data and the corresponding fit parameters are mentioned in the box below.

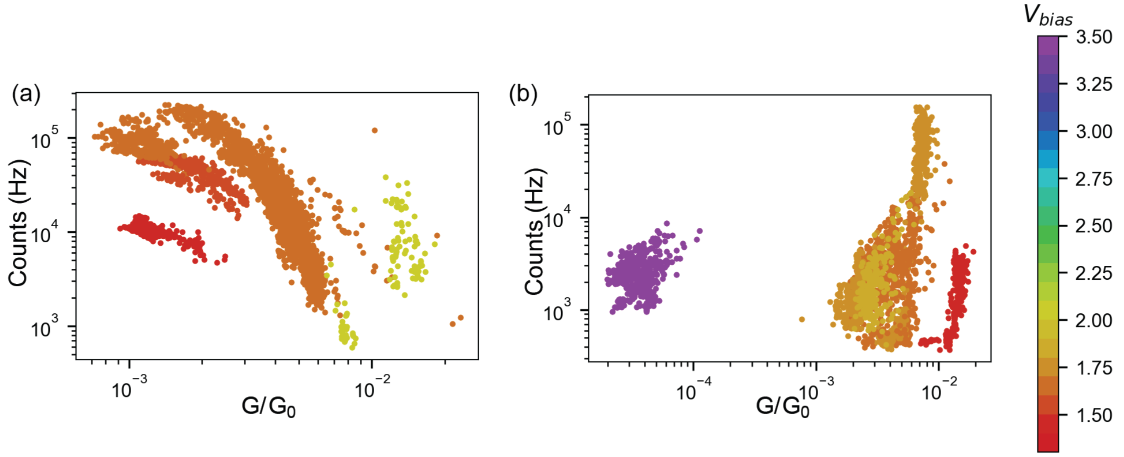

Figure S23: Correlation between photon count rate and conductance measured on a few other citrate-spaced PMJs. Each plot from (a)-(b) represents an individual PMJ. The different colors indicate the corresponding applied DC bias voltages.

S10 Picocavity events in SERS and conductance

To show evidence of picocavity formation during intermittent blinking, we performed a combined conductance and SERS measurement on our PMJ. The picocavities in the nanogap create strong optical field gradients that modify the Raman selection rules and create additional vibrational peaks in the SERS spectra.[S12] In Fig. S24, we show a few picocavity events from PMJs that are correlated with the conductance jumps.

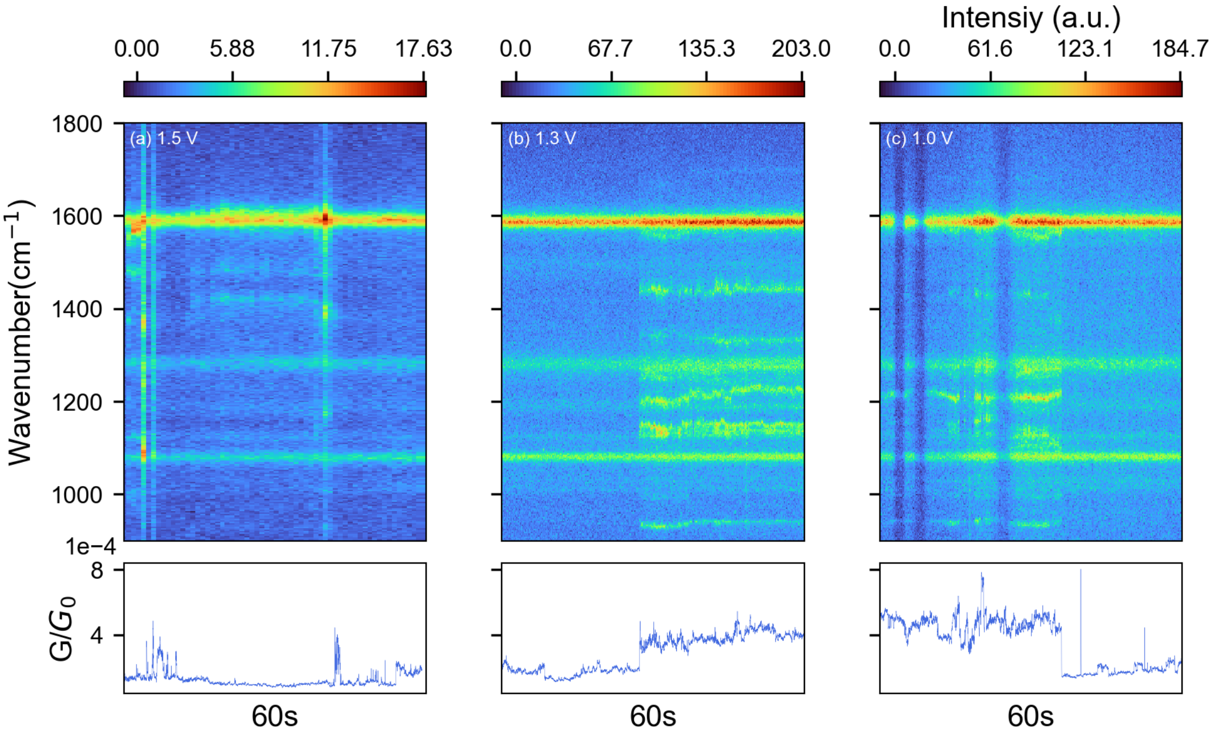

Figure S24: Time series of SERS spectrum along with the conductance showing picocavity events.

Such correlated events are very rare in SAM molecular junctions because we need to capture the conductance and SERS signal from the same molecule to observe the correlation. SERS in general is a collective signal obtained from all the molecules present in the nanogap, whereas conductance is obtained from a very few molecules connecting the electrodes. Thus the Raman signal is dominated by several other molecules that are not involved in the transport. Even in the presence of picocavities, it needs not occur from the same molecule that transmits the electrons. Such events are much less probable in PMJ as opposed to break junctions with atomic-sized tips.[S13] PMJs with single nanoparticle devices could be useful to perform such combined SERS and conductance measurements, where there will be better chances to observe direct evidence of picocavity events in transport.

S11 Outlook: Formation of single nanoparticle junction

To trap a single nanoparticle in the nanogap, the dielectrophoresis (DEP) technique with feedback from optical imaging is used. DEP involves controlling a polarizable object with a non-uniform electric field.[S14] When a particle is exposed to a non-uniform electric field, it is polarized and builds its own induced dipole moment. The DEP forces steer the particle towards the region of higher field intensity. The DEP force experienced by a spherical nanoparticle depends on several factors including the field intensity variation, the surrounding medium, and the size of the particle. Hence, DEP can be used to trap nanoparticles of varied concentration by applying an oscillating voltage of varied magnitude and frequency. The DEP forces can be expressed by the equation,

$$< \mathbf{F}_{DEP}(\omega) >= 2\pi\epsilon_m R^3 Re[f_{CM}(\omega)]\nabla|\mathbf{E}_{rms}|^2 \tag{6}$$

where ϵ_m is the permittivity of the medium, R is the radius of the nanoparticle, $f_{CM}(\omega)$ is the Clausius-Mosotti factor, and E_{rms} is the rms value of the electric field. The Clausius-Mosotti factor describes the complex polarizability of the particle and is given by

$$f_{CM}(\omega) = \frac{\epsilon_p - \epsilon_m - \frac{j}{\omega}(\sigma_p - \sigma_m)}{\epsilon_p + 2\epsilon_m - \frac{j}{\omega}(\sigma_p + 2\sigma_m)} \tag{7}$$

where ϵ_p and σ_p are the permittivity and the conductivity of the particle, while ϵ_m and σ_p correspond to that of the medium, $\omega = 2\pi f$ is the angular frequency.

For the trapping to occur, the DEP forces have to be larger than the thermal motion described by,

$$F_{th} = \frac{k_B T}{2R} \tag{8}$$

where k_B is the Boltzmann constant and T is the temperature.

We ideally want to trap single nanoparticles in the gap. It has been shown that one could control the concentration of nanoparticles, voltage amplitude, and frequency of the oscillating field, and time of application of the voltage to manipulate the number of particles in the gap.[S15–S19]

An optical microscope was modified to include probes that are attached to the sample holder (Fig. S25a). The probes can contact the chip directly for the DEP experiment. A water immersion objective is used to observe the dark-field image throughout the trapping process. About 50 μl of DI water is placed between the objective and the sample for imaging. 8 μl of 10 OD nanoparticle solution in DI water is added to the

solution. AC voltage between 1-3 V with a frequency of 500 kHz - 1 MHz is used to trap the nanoparticles. Different samples (normally with varied electrode widths and nanogap) require slightly different voltages and frequencies to trap the particles. The trapping can be monitored live from dark-field imaging. The DF image before and after trapping the nanoparticles is shown in Fig. S25b,c.

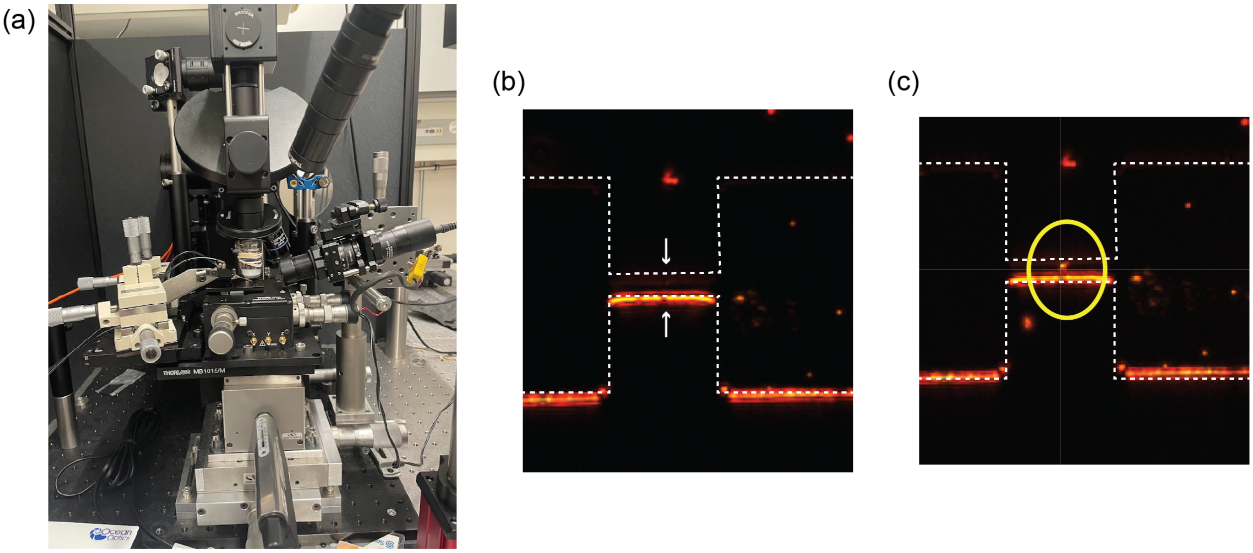

Figure S25: (a) Optical microscope with probes for DEP trapping. DF image of the electrode structures before (b) and after (c) trapping the nanoparticles by DEP. For better visibility, white dotted lines are overlayed on the DF image in (b,c) to mark the boundaries of the electrodes. White arrows point to the electrode gap. The yellow circle marks the location of the trapped particle.

The AC voltage is stopped once the particles are trapped, and the excess solution is blow-dried. Sometimes the particles are trapped with AC voltage but get released when the voltage is turned off. In such cases, a small DC voltage of 100 mV helps to stick the particles in the gap. With this approach, we could obtain devices with almost 100% yield. Sometimes, if the image was not clear enough due to excess scattering from the electrodes, we might end up with more than one nanoparticle. Otherwise, a single nanoparticle junction could be achieved reproducibly.

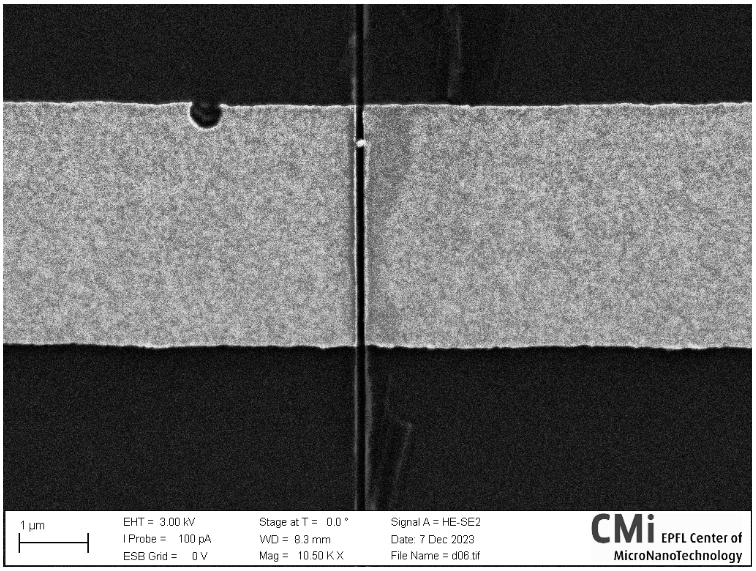

Figure S26: Single nanoparticle junction

When the electrodes are functionalized with BPDT molecules, it increases the natural deposition rate of the molecules. This is not suitable for optical measurements as many particles stick in the vicinity of the electrode gap. To avoid this, one could try molecular functionalization and nanoparticle deposition without removing the PMMA layer (e-beam resist).

Note: These are preliminary results. The devices used for experiments discussed in this manuscript are from junctions with a few nanoparticles bridging the electrodes as described in Sec. S1.2.

References

(S1) Ahmed, A.; Banjac, K.; Verlekar, S. S.; Cometto, F. P.; Lingenfelder, M.; Galland, C. Structural Order of the Molecular Adlayer Impacts the Stability of Nanoparticle-on-Mirror Plasmonic Cavities. *ACS Photonics* **2021**, *8*, 1863–1872.

(S2) Flauraud, V.; Mastrangeli, M.; Bernasconi, G. D.; Butet, J.; Alexander, D. T. L.; Shahrabi, E.; Martin, O. J. F.; Brugger, J. Nanoscale topographical control of capillary assembly of nanoparticles. *Nature Nanotechnology* **2017**, *12*, 73–80.

(S3) Guo, S.; Hihath, J.; Díez-Pérez, I.; Tao, N. Measurement and Statistical Analysis of Single-Molecule Current–Voltage Characteristics, Transition Voltage Spectroscopy,

and Tunneling Barrier Height. *Journal of the American Chemical Society* **2011**, *133*, 19189–19197.

(S4) Jeong, H.; Li, H. B.; Domulevicz, L.; Hihath, J. An On-Chip Break Junction System for Combined Single-Molecule Conductance and Raman Spectroscopies. *Advanced Functional Materials* **2020**, *30*, 2000615.

(S5) Ramachandran, R.; Li, H. B.; Lo, W.-Y.; Neshchadin, A.; Yu, L.; Hihath, J. An Electromechanical Approach to Understanding Binding Configurations in Single-Molecule Devices. *Nano Letters* **2018**, *18*, 6638–6644.

(S6) Domulevicz, L.; Jeong, H.; Paul, N. K.; Gomez-Diaz, J. S.; Hihath, J. Multidimensional Characterization of Single-Molecule Dynamics in a Plasmonic Nanocavity. *Angewandte Chemie International Edition* **2021**, *60*, 16436–16441.

(S7) Vonlanthen, D.; Mishchenko, A.; Elbing, M.; Neuburger, M.; Wandlowski, T.; Mayor, M. Chemically Controlled Conductivity: Torsion-Angle Dependence in a Single-Molecule Biphenyldithiol Junction. *Angewandte Chemie International Edition* **2009**, *48*, 8886–8890.

(S8) Mishchenko, A.; Vonlanthen, D.; Meded, V.; Bürkle, M.; Li, C.; Pobelov, I. V.; Bagrets, A.; Viljas, J. K.; Pauly, F.; Evers, F.; Mayor, M.; Wandlowski, T. Influence of Conformation on Conductance of Biphenyl-Dithiol Single-Molecule Contacts. *Nano Letters* **2010**, *10*, 156–163.

(S9) Jaklevic, R. C.; Lambe, J. Molecular Vibration Spectra by Electron Tunneling. *Physical Review Letters* **1966**, *17*, 1139–1140.

(S10) Chen, W.; Roelli, P.; Hu, H.; Verlekar, S.; Amirtharaj, S. P.; Barreda, A. I.; Kippenberg, T. J.; Kovylina, M.; Verhagen, E.; Martínez, A.; Galland, C. Continuous-wave frequency upconversion with a molecular optomechanical nanocavity. *Science* **2021**, *374*, 1264–1267.

(S11) Baumberg, J. J.; Aizpurua, J.; Mikkelsen, M. H.; Smith, D. R. Extreme nanophotonics from ultrathin metallic gaps. *Nature Materials* **2019**, *18*, 668–678.

(S12) Benz, F.; Schmidt, M. K.; Dreismann, A.; Chikkaraddy, R.; Zhang, Y.; Demetriadou, A.; Carnegie, C.; Ohadi, H.; Nijs, B. d.; Esteban, R.; Aizpurua, J.; Baumberg, J. J. Single-molecule optomechanics in "picocavities". *Science* **2016**, *354*, 726–729.

(S13) Ward, D. R.; Halas, N. J.; Ciszek, J. W.; Tour, J. M.; Wu, Y.; Nordlander, P.; Natelson, D. Simultaneous Measurements of Electronic Conduction and Raman Response in Molecular Junctions. *Nano Letters* **2008**, *8*, 919–924.

(S14) Pethig, R. Review Article—Dielectrophoresis: Status of the theory, technology, and applications. *Biomicrofluidics* **2010**, *4*, 022811.

(S15) Barsotti, R. J.; Vahey, M. D.; Wartena, R.; Chiang, Y.-M.; Voldman, J.; Stellacci, F. Assembly of Metal Nanoparticles into Nanogaps. *Small* **2007**, *3*, 488–499.

(S16) Yoon, S.-H.; Kumar, S.; Kim, G.-H.; Choi, Y.-S.; Kim, T. W.; Khondaker, S. I. Dielectrophoretic Assembly of Single Gold Nanoparticle into Nanogap Electrodes. *Journal of Nanoscience and Nanotechnology* **2008**, *8*, 3427–3433.

(S17) Gierhart, B. C.; Howitt, D. G.; Chen, S. J.; Smith, R. L.; Collins, S. D. Frequency Dependence of Gold Nanoparticle Superassembly by Dielectrophoresis. *Langmuir* **2007**, *23*, 12450–12456.

(S18) Kumar, S.; Yoon, S.-H.; Kim, G.-H. Bridging the nanogap electrodes with gold nanoparticles using dielectrophoresis technique. *Current Applied Physics* **2009**, *9*, 101–103.

(S19) Cheon, D.; Kumar, S.; Kim, G.-H. Assembly of gold nanoparticles of different diameters between nanogap electrodes. *Applied Physics Letters* **2010**, *96*, 013101.
